# An illustrated key to the genera of Eumeninae from China, with a checklist of species (Hymenoptera, Vespidae)

**DOI:** 10.3897/zookeys.740.22654

**Published:** 2018-03-02

**Affiliations:** 1 Shaanxi Key Laboratory for Animal Conservation/Key Laboratory of Resource Biology and Biotechnology in Western China, Ministry of Education, College of Life Sciences, Northwest University, Xi’an, China; 2 Division of Invertebrate Zoology, American Museum of Natural History, New York, NY, USA

**Keywords:** homonym, illustrated key, new record, Oriental, Palaearctic

## Abstract

An illustrated key to the currently recognized genera of the subfamily Eumeninae (Vespidae) from China is presented together with a list of 267 species and subspecies, belonging to 51 genera. *Nortozumia* van der Vecht, 1937 is reported for the first time from China. Two replacement names are proposed for junior primary homonyms: *Ancistrocerus rufofrustius* Tan & Carpenter, **nom. n.** replacing *Ancistrocerus rufopictus* (Kostylev) and *Orientalicesa confasciatus* Tan & Carpenter, **nom. n.** replacing *Orientalicesa unifasciatus* (von Schulthess, 1934).

## Introduction

Eumeninae or potter wasps are the largest subfamily of the Vespidae with 3773 valid species in 205 genera (Carpenter 1986; Zhou et al. 2011; Tan et al. 2015, 2018a; Pannure et al. 2016; Carpenter unpubl.). Eumeninae have a cosmopolitan distribution and are morphologically very diverse. The generic classification of Eumeninae is chaotic and has a troubled taxonomic history. The extreme splitting haphazardly pursued during much of the 20th century has contributed much to this current state (Hermes et al. 2014). Clearly, the situation with the generic classification will have to be rationalized by future synonymy of numerous taxa after their phylogeny is better known (Carpenter and Cumming 1985; Carpenter and Garcete-Barrett 2003; Hermes et al. 2014). The need for taxonomic work on Eumeninae is underlined by the lack of adequate and well-illustrated keys, both to genera and to species (Pannure et al. 2016). The few generic keys available concern one region or a country: Yamane (1990) revised the Japanese fauna of Eumeninae with a key to 18 genera, Carpenter and Garcete-Barrett (2003) presented a key to the genera of Neotropical Eumeninae and Pannure et al. (2016) included a key to the 31 eumenine genera known from South India. We present the first illustrated key to genera of Eumeninae from a major area encompassing two faunal regions and the first complete key to genera of Chinese Eumeninae. It is a major step to facilitate the classification of Chinese Eumeninae. Nevertheless, the status of several genera remains problematical; only a combined approach using molecular, biological, and morphological data will make it possible to decide their taxonomic position.

The Chinese Eumeninae were first catalogued by Liu (1936–1937) resulting in a list of 57 species divided among nine genera. Unfortunately, his research stopped after his only revision (*Pareumenes*; Liu 1941). Lee (1982a, 1985) published the most recent key to the genera of Eumeninae in China, including only 25 genera (for 65 species and 13 subspecies). Finally, Zhou et al. (2011) listed 45 genera present in China and included 172 species and 50 subspecies. Several scattered papers have been published on Chinese Eumeninae, but a thorough inventory is lacking (Zhou et al. 2012, 2013; Li and Chen 2014a, 2014b, 2015, 2016a, 2016b; You et al. 2013; Ma et al. 2016, 2017; Nguyen and Xu 2014; 2015; 2017; Yeh and Lu 2017; Tan et al. 2018a). The illustrated key to the genera of Chinese Eumeninae presented here includes 51 genera and the checklist contains 267 species and subspecies in total. One genus (*Nortozumia* van der Vecht, 1937) is reported as new to China. Two replacement names are proposed for junior primary homonyms: *Ancistrocerus rufofrustius* Tan & Carpenter, nom. n. replacing *Ancistrocerus rufopictus* (Kostylev) and *Orientalicesa confasciatus* Tan & Carpenter, nom. n. replacing *Orientalicesa unifasciatus* (von Schulthess, 1934).

## Material and methods

Specimens were collected by hand net or with Malaise traps. The studied specimens are deposited in the collections of College of Life Sciences, Northwest University, Xi’an (**NWUX**); Northwest A&F University Entomological Museum, Yangling, Shaanxi (**NWAY**); Zhejiang University Hymenoptera Museum, Hangzhou (**ZJUH**); General Station of Forest Pest Management, State Forestry Administration, Shenyang (**GSFA**); American Museum of Natural History, New York (**AMNH**); Naturalis Biodiversity Center, Leiden (**RMNH**); Museum national d’Histoire naturelle, Paris (**MNHN**); and Senckenberg Deutsches Entomologisches Institut, Müncheberg (**SDEI**).

Morphological terminology follows Carpenter and Cumming (1985), Yamane (1990), and Carpenter and Garcete-Barrett (2003). Observations and descriptions were made with an Olympus SZX11 stereomicroscope and fluorescent lamps. Photographic images were made with Keyence VHX-5000 digital microscope (NWUX, Xi’an), Olympus SZX 12 stereomicroscope with analySIS Soft Imaging System software (RMNH, Leiden), and Microptics-USA/Visionary Digital photomicrographic system, developed by Roy Larimer, multiple layers stacked using Helicon Focus (AMNH, New York).

### Key to genera of Eumeninae from China

**Table:** 

1	Tergum I more or less petiolate, slender and its apical half parallel-sided or slightly narrowed posteriorly (1a); tergum I usually at least twice as long as wide (but in e.g., *Pseudozumia* slightly longer than wide); tergum II twice maximum width of tergum I in dorsal view, but approx. 1.5 × in *Pseudozumia*	**2**
–	Tergum I robust and its apical half widened posteriorly (1aa); tergum I much less than twice as long as wide; tergum II at most twice as wide as tergum I	**18**
2	Middle tibia with two spurs (2a)	**3**
–	Middle tibia with one spur (2aa)	**5**
3	Propodeum with valvula distinctly protruding, elongate and rectangular (3a), orifice acutely pointed dorsally (3b)	***Zethus* Fabricius**
–	Propodeum with valvula not protruding (3aa), orifice rounded dorsally (3bb)	**4**
4	Maxillary palpus 3-segmented (4a); tergum I more than twice as long as wide, longitudinally striate (4b); tegula posteriorly acute (4c)	***Calligaster* de Saussure**
–	Maxillary palpus 4-segmented (4aa); tergum I less than twice as long as wide, punctate (4bb); tegula posteriorly truncate (4cc)	***Discoelius* Latreille**
5	Propodeal valvula relatively short, rounded, submarginal carina distinctly protruding (5a); frons distinctly longer than clypeus; second submarginal cell narrow anteriorly, vein 1-M and vein 2-M meeting almost at right angle (5b)	**6**
–	Propodeal valvula elongate, rectangular, submarginal carina not protruding (4aa); frons shorter than clypeus; second submarginal cell wide anteriorly, vein 1-M and vein 2-M meeting at obtuse angle (5bb)	**8**
6	Female with fovea anterior to mid-ocellus (6a); metanotum monodentiform mesally (6d); metasomal petiole abruptly swollen apically in lateral view (6c); tegula not exceeding parategula posteriorly (6b); anterior face of pronotum smooth (6e)	***Labus* de Saussure**
–	Female without fovea on vertex (6aa); metanotum obtuse mesally (6dd); metasomal petiole not conspicuously swollen in lateral view (6cc); tegula more or less exceeding parategula posteriorly (6bb); anterior face of pronotum distinctly punctuate laterally (6ee)	**7**
7	Mesoscutum distinctly longer than wide (7a), apical margin of tergum I wider than half width of TII (7b); parategula short, almost absent, tegula distinctly exceeding parategula (7c)	***Leptomicrodynerus* Giordani Soika**
–	Mesoscutum distinctly wider than long (7aa); apical margin of tergum I narrower than half width of tergum II (7bb); parategula normal, tegula slightly exceeding parategula (7cc)	***Cyrtolabulus* van der Vecht**
8	Propodeum dorsally with elongate fovea from which a carina runs to orifice, usually with dentiform projections above valvulae (8a); axillary fossa narrower than long, slit-like (8b); tegula with narrow posterior lobe which about equals parategula posteriorly (8c)	**9**
–	Propodeum without fovea or dentiform projections (8aa); axillary fossa oval, broader than long (8bb); tegula short, convex and not equalling parategula posteriorly (8cc), or long, with narrow posterior lobe which surpasses parategula posteriorly	**14**
9	Mesepisternum with epicnemial carina present (9a)	**10**
–	Mesepisternum with epicnemial carina absent (9aa)	**13**
10	Metasomal petiole with transverse carina basally (10a, a’)	**11**
–	Metasomal petiole not carinate (10aa, aa’)	**12**
11	Tergum I with its lateral margins fused ventrally; sternum I reduced to posterior crescentic sclerite (11a); [mesonotum and propodeum smooth between fine punctures]	***Ectopioglossa* Perkins**
–	Tergum I with lateral margins not meeting ventrally, sternum I visible along entire petiole length (11aa)	***Nortozumia* van der Vecht** (new record)
12	Mesoscutum with a pair of prescutellar longitudinal groove (12a); forewing with parastigma longer than pterostigma (12b); sternum I irregularly rugose posteriorly, with rugae running in longitudinal direction (12c)	***Pseudozumia* de Saussure**
–	Mesoscutum without prescutellar longitudinal groove (12aa); forewing with parastigma shorter than pterostigma (12bb); sternum I smooth basally, its posterior two thirds transversely striate or smooth (12cc)	***Coeleumenes* van der Vecht**
13	Forewing with parastigma longer than pterostigma (13a); sternum I gradually widened backwards with regular transverse striae (13b); female with cephalic fovea (13c); hind tibia with number of short spines on its outside (13d)	***Pareumenes* de Saussure**
–	Forewing with parastigma shorter than pterostigma (13aa); sternum I narrower basally, more or less fused with tergum I, posteriorly short, triangular and without ruga (13bb); female without cephalic fovea (13cc); hind tibia without spines on its outside (13dd)	***Pseumenes* Giordani Soika**
14	Tergum I impunctate or with only a few small punctures (14c); propodeum inclining posteriorly into a slope (14a); tergum II without lamella separated by preapical thickening, sometimes with pale border (14b)	**15**
–	Tergum I with dense, coarse punctation (14cc); propodeum less inclined posteriorly (14aa); tergum II with apical lamella more or less separated from disc by preapical thickening (14bb)	**17**
15	Clypeus apically rounded (15a); temple in dorsal view as long as eye (15b)	***Katamenes* Meade-Waldo**
–	Clypeus apically truncate or emarginated (15aa); temple in dorsal view shorter than eye (15bb)	**16**
16	Tergum I slightly longer than mesosoma, with section after spiracles shorter than section before spiracles (16a); male: terminal sternum with a longitudinal groove (16b)	***Delta* de Saussure**
–	Tergum I much longer than mesosoma, with section after spiracles longer than section before spiracles (16aa); male: terminal sternum without groove (16bb)	***Phimenes* Giordani Soika**
17	Pronotum with pretegular carina absent (17b); parastigma of forewing shorter than half of pterostigma (17a); female: apical margin of clypeus emarginated (17c); propodeum lateral margin rounded, without distinct border with posterior face; male: apical antennal segment medium-sized and curved (17d)	***Eumenes* Latreille**
–	Pronotum with pretegular carina present (17bb); parastigma of forewing longer than half of pterostigma (17aa); female: apical margin of clypeus truncated (17cc); propodeum lateral side distinctly separated from its posterior face; male: apical antennal segment small and straight (17dd)	***Oreumenes* Bequaert**
18	Tegula evenly rounded posteriorly and usually not reaching apex of parategula (18a); male antenna apically spiralled (18b)	**19**
–	Tegula protruding posteriorly, emarginate or truncate adjoining parategula (18aa); male antenna apically hooked or simple (18bb)	**22**
19	Labial palpi 3-segmented, in female segment II and III both broadly flattened, fringed with setae, forming a psammophore (19a); sterna II-V in male usually with central apical brush (19b)	**20**
–	Labial palpus 4-segmented, in female cylindrical, without psammophore (19aa); sterna II-V in male usually without central apical brush (19bb)	**21**
20	Maxillary palpus 5- (20a) or 6- (20a’) segmented; female: labial palpus segment II thick basally, segment II, III not curved (20b); male: mandible with penultimate tooth often without deep excision, and axis of penultimate tooth is at an oblique angle relative to main axis of mandible and approximately parallel to axis of apical tooth (20c); body <9 mm	***Pterocheilus* Klug**
–	Maxillary palpus 6-segmented (20aa); female: labial palpus segment II and III slender, flat and curved (20bb); male: mandible with distance between second and third tooth broad (20cc) or deeply and broadly excised, and axis of penultimate tooth is at approximately a right angle relative to the main axis of the mandible and relative to the apical tooth (20cc’); body >10 mm	***Onychopterocheilus* Blüthgen**
21	Propodeum with lateral carinae well developed (21a); female without cephalic foveae (21b); vertex weakly longitudinally rugose posteriorly (21b); temples and mesosoma with very fine pubescence; male sterna II-VI with conspicuous fringe of setae (21c)	***Tropidodynerus* Blüthgen**
–	Propodeum with lateral carinae weak or absent (21aa); female with cephalic foveae; vertex not rugose; temples and mesosoma with long setae (21bb); male sterna without fringe of setae (21cc)	***Odynerus* Latreille**
22	Anterior face of pronotum with two close and deeply impressed pits, which may be approximated, or with series of elongate foveae (22a); tegula broad, wider than long, not surpassing parategula (except *Jucancistrocerus*) (22b); propodeum valvula bilamellate (with submarginal carina produced into pointed lamella apically and valvula enlarged and free posteriorly from submarginal carina) (22c)	**23**
–	Anterior face of pronotum without deep pits or foveae (except punctures) (22aa, aa’); submarginal carina, propodeum valvula and tegula variable	**28**
23	Tergum I with two transverse carinae (23a) or with one (23a’)	**24**
–	Tergum I without transverse carinae (23aa)	**27**
24	Tegula densely punctate, sieve-like, surpassing parategula posteriorly (24a); propodeal dorsum without extending horizontal area (24b); pretegular carina absent (24c); [carina of tergum I indistinct in some species]	***Jucancistrocerus* Blüthgen**
–	Tegula usually finely punctate (24aa); propodeal dorsum extending horizontally, forming shelf-like area behind metanotum (24bb); pretegular carina present (24cc)	**25**
25	Tergum I with two carinae, tergum wider than long in dorsal view, both carinae relatively close near each other (25a)	***Subancistrocerus* de Saussure**
–	Tergum I with one (25aa) or two carinae (25aa’); **if** with two carinae, then tergum I longer than wide in dorsal view, more or less petiole-like and distance between both carinae relatively large	**26**
26	Anterior face of pronotum with foveae separated (26a); tergum II usually smooth basally, forming an acarinarium (26b); metasoma sessile, tergum I nearly as wide as tergum II (26b’)	***Parancistrocerus* Bequaert**
–	Anterior face of pronotum with contiguous foveae (26aa); tergum II ridged basally, not forming an acarinarium; tergum I in dorsal view longer than wide, tergum II much wider than tergum I (26bb)	***Pseudonortonia* Giordani Soika**
27	Tergum I relatively short, gradually widened, with its lateral sides divergent in dorsal view (27a); vertex strongly depressed, forming an acute triangle with face (27b, arrowing part); in female, apical margin of clypeus truncated and with two longitudinal carinae (27c); anterior face of pronotum with foveae coalesced (27b)	***Paraleptomenes* Giordani Soika**
–	Tergum I relatively long, roughly parallel-sided in dorsal view (27aa); vertex normal, not forming an acute triangle with face (27bb); apical margin of clypeus emarginate and without carina (27cc); anterior face of pronotum with foveae separated (22a). [Note: if tergum I of *Parancistrocerus* spp. has an indistinct transverse carina, then it is difficult to separate them from *Stenodynerus*; *Parancistrocerus* spp. usually have an acarinarium on tergum II basally and tergum I more or less lengthened medially in dorsal view, while *Stenodynerus* spp. have tergum II ridged basally and tergum I medio-dorsally without elongation]	***Stenodynerus* de Saussure**
28	Tergum I transversely carinate (28a) **or** horizontal and vertical faces of tergum clearly separated (*Pararrhynchium*, 28a’)	**29**
–	Tergum I evenly curved, without transverse carina (28aa)	**34**
29	Tergum I with broad medio-longitudinal furrow posterior to transverse carina (29a); notauli clearly indicated (29b); male antenna simple apically (29c)	***Symmorphus* Wesmael**
–	Tergum I without medio-longitudinal furrow (29aa); notauli absent or nearly so (29bb); male antenna hooked apically (29cc)	**30**
30	Parastigma of forewing with more than half the length of pterostigma, measured along posterior part, often nearly equal (30a)	**31**
–	Parastigma half the length of pterostigma or less, measured along posterior part (30aa)	**33**
31	Tergum II with well-developed apical lamella (31a)	***Lissodynerus* Giordani Soika**
–	Tergum II lacking an apical lamella (31aa)	**32**
32	Clypeus wide ventrally and slightly emarginated medio-ventrally (32a); male: terminal sternum without teeth basally (32b)	***Orancistrocerus* van der Vecht**
–	Clypeus narrower ventrally and deeply emarginated medio-ventrally (32aa); male: terminal sternum with 2–3 teeth basally (32bb)	***Archancistrocerus* Giordani Soika**
33	Axillary fossa narrower than long, slit-like (33b); humeri (“shoulder”) rounded (33a); propodeal dorsum strongly extending horizontally, forming shelf-like area behind metanotum (33c)	***Pararrhynchium* de Saussure**
–	Axillary fossa oval, broader than long (33bb); humeri angular or pointed (33aa); propodeal dorsum slightly extending behind metanotum, below level of metanotum (33cc)	***Ancistrocerus* Wesmael**
34	Metanotum with serrate bilobed ridge (34a, a’)	**35**
–	Metanotum low toothed (34aa), including blunt or rounded off ridge or flat	**36**
35	Clypeus higher than wide (35a); metanotum with truncate teeth; mid-anterior face of pronotum smooth and with short transverse rugae (35b); tergum I distinctly narrower than tergum II (35c); male terminal antennal segment small (35d)	***Apodynerus* Giordani Soika**
–	Clypeus wider than high (35aa); mid-anterior face of pronotum usually densely punctate and with an upper trace of transverse carina (35bb); tergum I slightly narrower than tergum II (35cc); male terminal antennal segment relatively large (35dd)	***Antepipona* de Saussure**
36	Tergum II with lamella behind transverse band (36a)	**37**
–	Tergum II without lamella behind transverse band, at most with narrow border (36aa). Note: few *Euodynerus* spp. may possess a distinct lamella on tergum II (see 45a), they can be separated from *Leptochilus* by having tergum I not depressed subapically (depressed in *Leptochilus*); and differs from *Gribodia*, *Stenodynerellus* and *Epsilon* by having oval axillary fossa, broader than long (slit-like, narrower than long in *Gribodia*, *Stenodynerellus* and *Epsilon*)	**40**
37	Tergum I depressed subapically, gradually widened with lateral sides divergent in dorsal view (37 aa); propodeum with submarginal carina projecting as rounded lobe above valvula, bilamellate (37bb); epicnemial carina absent (37cc); axillary fossa oval, broader than long (37dd)	***Leptochilus* de Saussure**
–	Tergum I not depressed subapically, usually with lateral sides roughly parallel in dorsal view (37aa); propodeum with submarginal carina not differentiated from valvula, mono-lamellate (37bb; except *Epsilon*); epicnemial carina present (37cc); axillary fossa narrower than long, slit-like (37dd)	**38**
38	Palpal formula 5:3 (38a); male vertex sometimes with large and deep depression (38b); propodeum without shelf-like protruding part and with lateral carinae well developed (38c); [metanotum angulated, second submarginal cell with second recurrent vein nearly or completely interstitial with third submarginal cell; terga I-V each with apical lamella]	***Gribodia* Zavattari**
–	Palpal formula 6:4 (38aa); male vertex without large and deep depression (38bb); propodeum with shelf-like protruding part (38cc) or absent (38cc’)	**39**
39	Clypeus longer than wide, apical margin usually truncate (39a); propodeum with submarginal carina not differentiated from valvula (39b); propodeum usually with dorsal surface at about same level as metanotum, lateral margin rounded; metanotum usually smoothly convex (39c); terga I-V each with apical lamella (39d); second submarginal cell with second recurrent vein variable	***Stenodyneriellus* Giordani Soika**
–	Clypeus longer than wide, apical margin emarginate (39aa); propodeum with submarginal carina projecting as rounded lobe above valvula (39bb); propodeum without raised shelf-like part, lateral margin carinate; metanotum angulated (39cc); only tergum II with lamella (39dd); second submarginal cell with second recurrent vein nearly or completely interstitial with third submarginal cell (39ee)	***Epsilon* de Saussure**
40	Axillary fossa in dorsal view much narrower than long, often slit-like (40a); tegula short, not exceeding parategula	**41**
–	Axillary fossa in dorsal view not slit-like, at least as wide as long, oval (40aa); tegula usually equal to or exceeding parategula	**45**
41	Propodeum with raised shelf-like part nearly at level of metanotum, metanotum flat (41a)	**42**
–	Propodeum below level of metanotum, metanotum various (41aa)	**43**
42	Forewing with third submarginal cell separated from apex of marginal cell by about half its length (42a); male sternum VII with basal truncate process (42b)	***Allorhynchium* van der Vecht**
–	Forewing with third submarginal cell separated from apex of marginal cell by about its own length (42aa); male sternum VII without process (42bb)	***Orientalicesa* Koçak and Kemal**
43	Parastigma of forewing more than half length of pterostigma, measured along posterior part or nearly equal (43a); palpal formula 6:4 (38aa)	**44**
–	Parastigma shorter than half length of pterostigma, measured along posterior part (43aa); palpal formula 5:3 (38a)	***Okinawepipona* Yamane**
44	Mesoscutum posteriorly and scutellum smooth, very sparsely and finely punctate (44a); metanotum depressed medially (44b); male middle femur basally emarginated (44c)	***Rhynchium* Spinola**
–	Mesoscutum and scutellum richly punctate throughout (44aa); metanotum not depressed medially (44bb); male middle femur not basally emarginate (44cc)	***Anterhynchium* de Saussure**
45	Tergum I behind apical band with well-developed lamella (45a)	**46**
–	Tergum I behind apical band without well-developed lamella (45aa)	**47**
46	Metanotum between horizontal and vertical area with hemi-circular carina (46a)	***Antodynerus* de Saussure**
–	Metanotum between areas without hemi-circular shaped carina (46aa)	***Euodynerus* Dalla Torre**
47	Pretegular carina absent (47a)	**48**
–	Pretegular carina present, at least posteriorly (47aa)	**49**
48	Tegula sparsely punctate, slightly exceeding parategula and much smaller than scutellum (48a); mid-anterior face of pronotum smooth (48b); metanotum with lateral lamellae (48c); female with single cephalic fovea situated near occipital carina (48d)	***Parodontodynerus* Blüthgen**
–	Tegula densely punctate, distinctly exceeding parategula, almost as large as scutellum (48aa); mid-anterior face of pronotum densely punctate (48bb); metanotum without lateral lamellae (48cc); female with single cephalic fovea situated halfway posterior ocelli and occipital carina (48dd)	***Brachyodynerus* Blüthgen**
49	Tegula narrower and longer, surpassing parategula posteriorly (49a). [female vertex with reniform fovea, about as wide as ocellar triangle; hind coxa with ventral lobes]	***Allodynerus* Blüthgen**
–	Tegula broad, equal to parategula posteriorly (49aa)	**50**
50	Propodeal valvula mono-lamellate (50a), with transverse carina, and with a dentate ridge laterally (50b); [metanotum ridge roughly bidentate-shaped]	***Pseudepipona* de Saussure**
–	Propodeal valvula bilamellate (50aa), without transverse carina, and without dentate ridge laterally; [small species (6 mm body length); anterior face of pronotum smooth; vertex with very small pits]	***Asiodynerus* Kurzenko**

**Figure 1.**
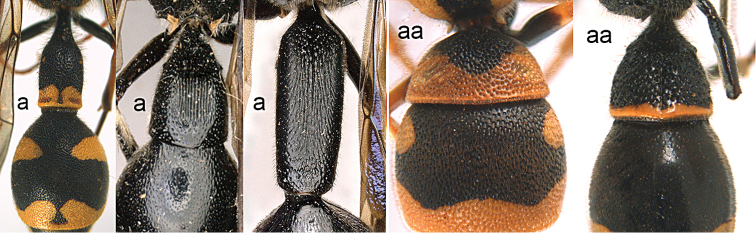
Metasomal terga I-II, dorsal view: **a** *Eumenes m. mediterraneus* (Kriechbaumer) (left 1), *Pseudozumia indica* (de Saussure) (left 2), *Calligaster cyanoptera* de Saussure (middle) **aa** *Antepipona deflenda lepeletieri* (Blüthgen) (right 2), *Symmorphus foveolatus* (Gussakovskij) (right1).

**Figure 2.**
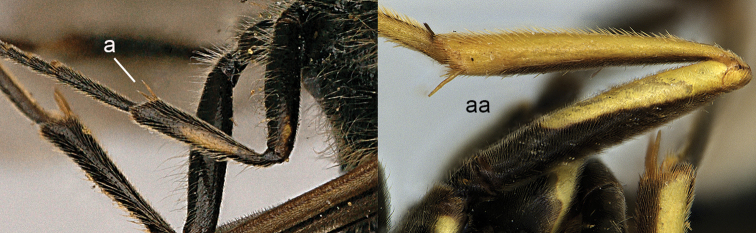
Part of middle leg: **a** *Discoelis zonalis* (Panzer) **aa** *Pseumenes depressus annulatus* van der Vecht.

**Figure 3.**
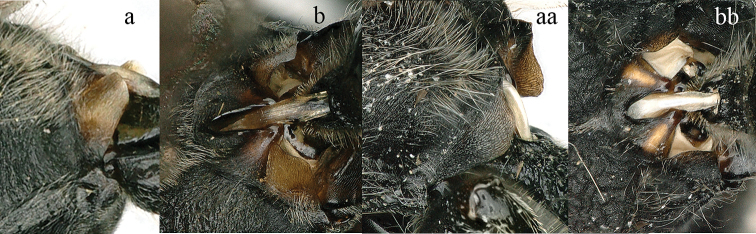
Part of propodeum lateral view (**a, aa**) and caudal view (**b, bb**): **a, b** *Zethus velamellatus* Tan **aa, bb** *Discoelis zonalis* (Panzer).

**Figure 4.**
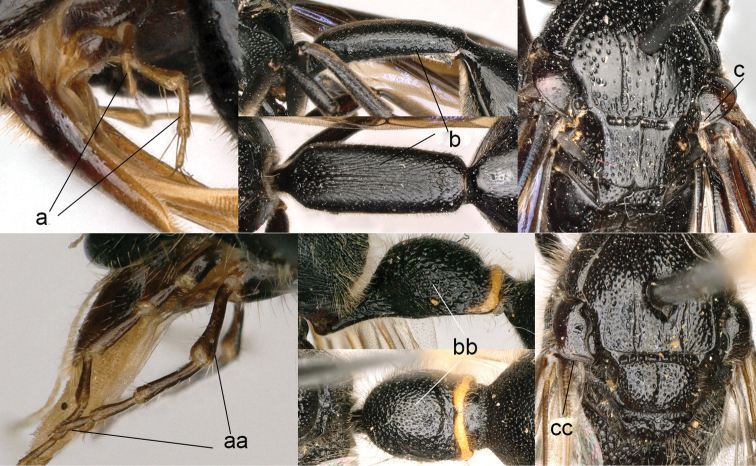
Labial palpi (**a, aa**) and metasomal segment I (**b, bb**): **c, cc** part of mesosoma, showing tegula: **a–c** *Calligaster cyanoptera* de Saussure **aa–cc** *Discoelis zonalis* (Panzer).

**Figure 5.**
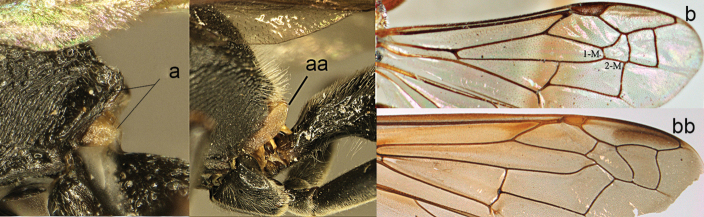
Propodeum (**a, aa**) and part of forewing (**b, bb**): **a** *Leptomicrodynerus tieshengi* Giordani Soika **b** *Labus spiniger* de Saussure (right upper) **aa** *Coeleumenes* sp. (middle) **bb** *Delta campaniforme esuriens* (Fabricius).

**Figure 6.**
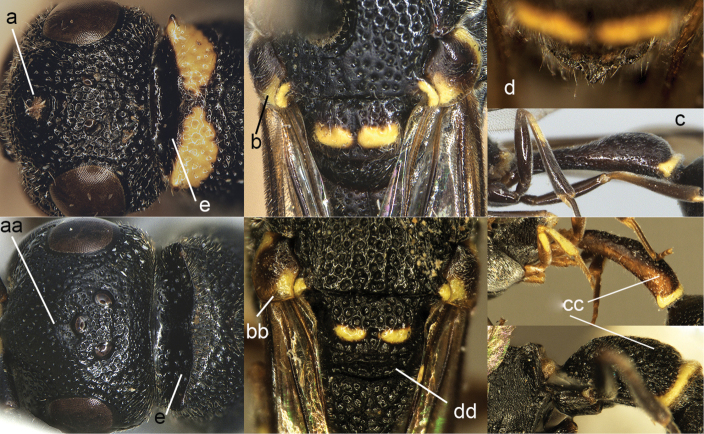
Head and pronotum (**a, e, aa, ee**), part of mesonotum in dorsal view (**b, bb, dd**), metanotum magnified (**d**), and metasomal segment I in lateral view (**c, cc**): **a–e** *Labus spiniger* (de Saussure) **aa, ee, lower cc** *Leptomicrodynerus tieshengi* Giordani Soika **bb, dd, upper cc** *Cyrtolabulus suavis* van der Vecht.

**Figure 7.**
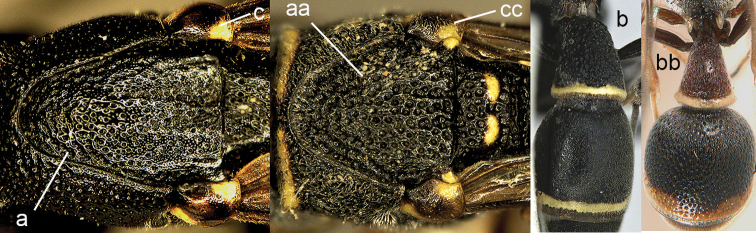
Mesosoma (**a, c, aa, cc**) and metasoma (**b, bb**). **a–c** *Leptomicrodynerus tieshengi* Giordani Soika **aa, cc** *Cyrtolabulus suavis* van der Vecht **bb** *Cyrtolabulus exiguus* (de Saussure).

**Figure 8.**
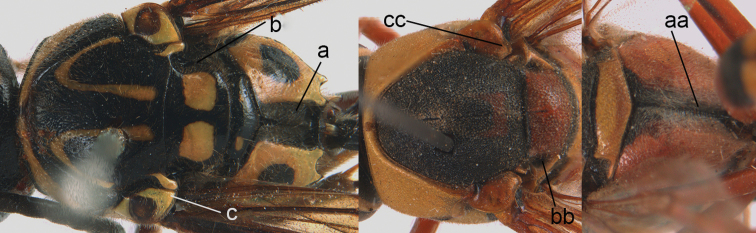
Mesosoma, dorsal view. **a–c** *Pseumenes d. depressus* (de Saussure) **aa–cc** *Delta campaniforme gracile* (de Saussure).

**Figure 9.**
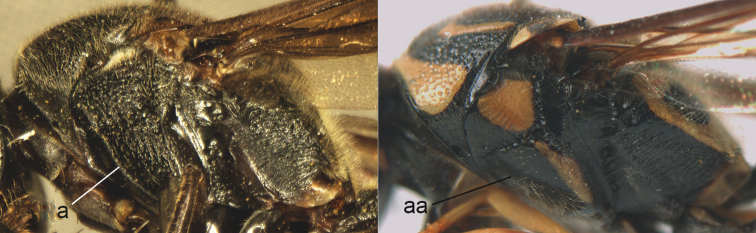
Mesosoma, lateral view. **a** *Nortozumia* sp. **aa** *Pseumenes d. depressus* (de Saussure).

**Figure 10.**
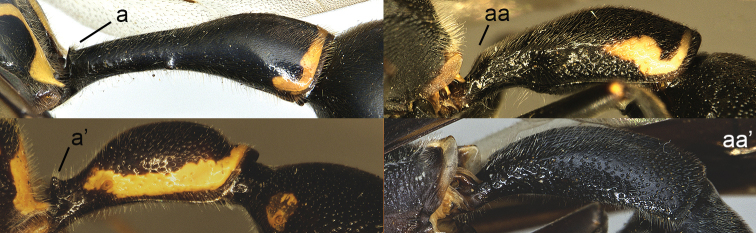
Metasomal segment I in lateral view. **a** *Ectopioglossa* *s. samariensis* (Giordani Soika) **a**’ *Nortozumia pulchella* (Smith) **aa** *Coeleumenes* sp. **aa**’ *Pseudozumia indica paulonotata* Giordani Soika.

**Figure 11.**
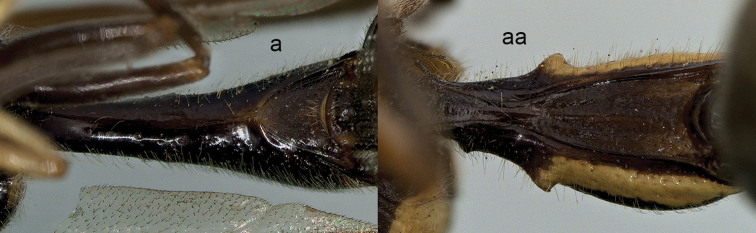
Metasomal segment I, ventral view. **a** *Ectopioglossa* *s. samariensis* (Giordani Soika) **aa** *Nortozumia pulchella* (Smith).

**Figure 12.**
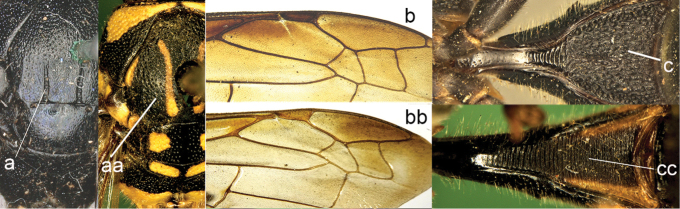
Part of dorsal mesosoma (**a, aa**), part of forewing (**b, bb**) and ventral metasomal segment I (**c, cc**). **a** *Pseudozumia i. indica* (de Saussure) **b** *Pseudozumia indica borneana* Giordani Soika **c** *Pseudozumia* sp. **aa–cc** *Coeleumenes impavidus* (Bingham).

**Figure 13.**
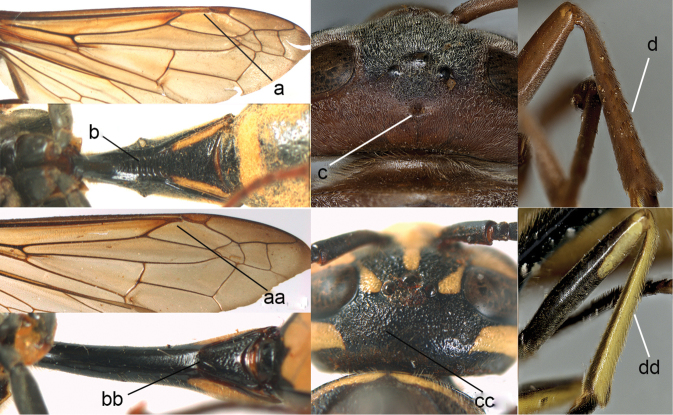
Forewing (**a, aa**), metasomal sternum I (SI) (**b, bb**), head in dorsal view (**c, cc**) and hind tibia (**d, dd**). **a–b** *Pareumenes quadrispinosus conjunctus* Liu **c, d** *Pareumenes* *s. sansibaricus* (von Schulthess) **aa–cc** *Pseumenes d. depressus* (de Saussure); dd. *Pseumenes depressus annulatus* van der Vecht.

**Figure 14.**
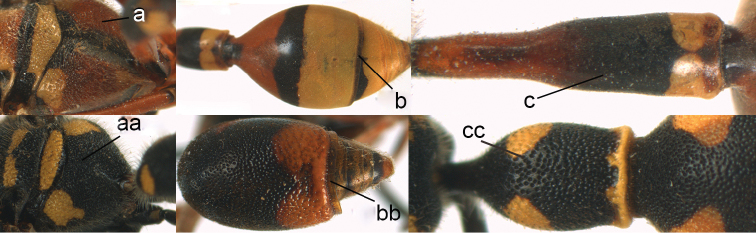
Propodeum (**a, aa**), metasomal tergum II (TII) (**b, bb**) and metasomal tergum I (TI) (**c, cc**). **a–c** *Delta campaniforme esuriens* (Fabricius) **aa, cc** *Eumenes c. coarctatus* (Linnaeus) **bb** *Eumenes kiangsuensis* Giordani Soika.

**Figure 15.**
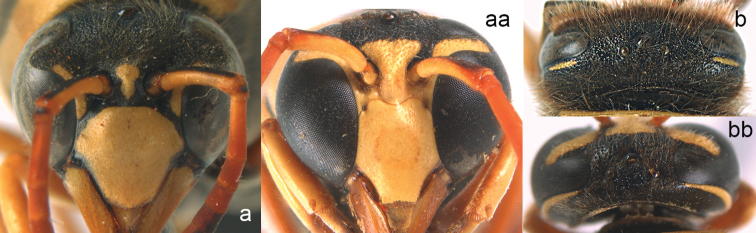
Head in frontal (**a, aa**) and dorsal view (**b, bb**). **a, b** *Katamenes sesquicinctus* (Lichtenstein) **aa, bb** *Delta campaniforme gracile* (de Saussure).

**Figure 16.**
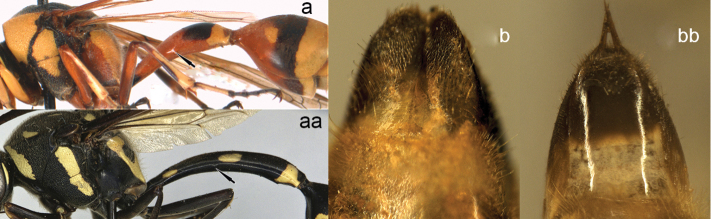
Mesosoma and part of metasoma (**a, aa**), terminal sternum of male (**b, bb**). **a, b** *Delta campaniforme gracile* (de Saussure), arrow = spiracle **aa, bb** *Phimenes f. flavopictus* (Blanchard).

**Figure 17.**
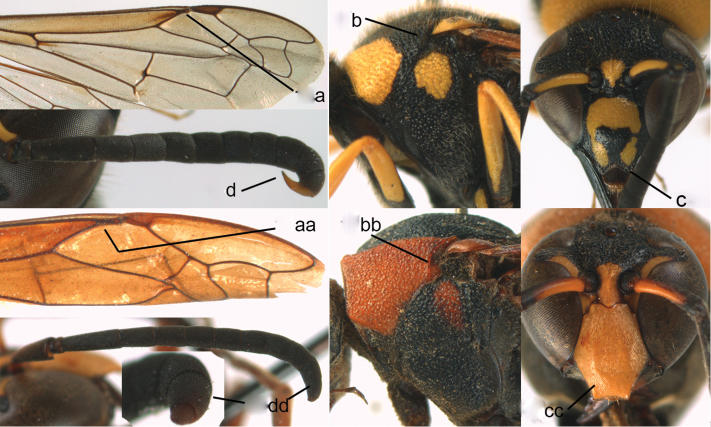
Forewing (**a, aa**), mesosoma in lateral view (**b, bb**), head in frontal view (**c, cc**) and antenna (**d, dd**). **a–d** *Eumenes c. coarctatus* (Linnaeus) **aa–dd** *Oreumenes decoratus* (Smith).

**Figure 18.**
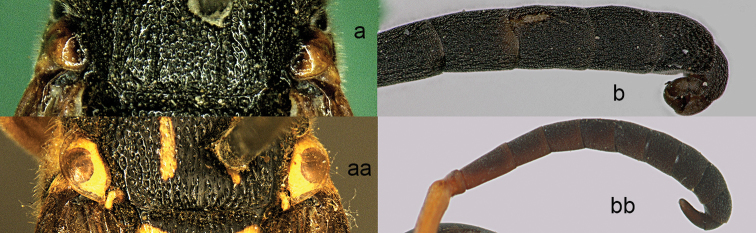
Part of mesosoma (**a, aa**) and antennae (**b, bb**). **a** *Onychopterocheilus mochii* (Giordani Soika) **b** *Pterocheilus p. phaleratus* (Panzer) **aa** *Stenodyneriellus guttulatus* (de Saussure) **bb** *Euodynerus d. dantici* (Rossi).

**Figure 19.**
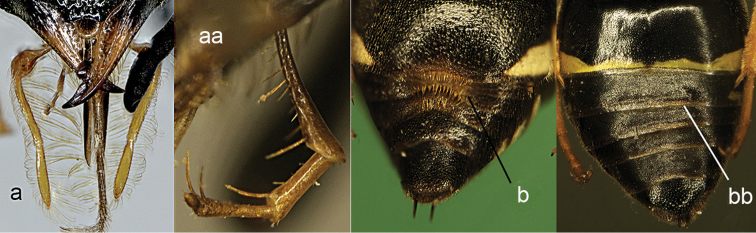
Part of mouthparts showing labial palpus (**a, aa**) and metasomal sterna (SII-VII) of male (**b, bb**). **a** *Onychopterochilus mochii* (Giordani Soika) **b** *Pterocheilus p. phaleratus* (Panzer) **aa–bb** *Odynerus albopictus* (de Saussure).

**Figure 20.**
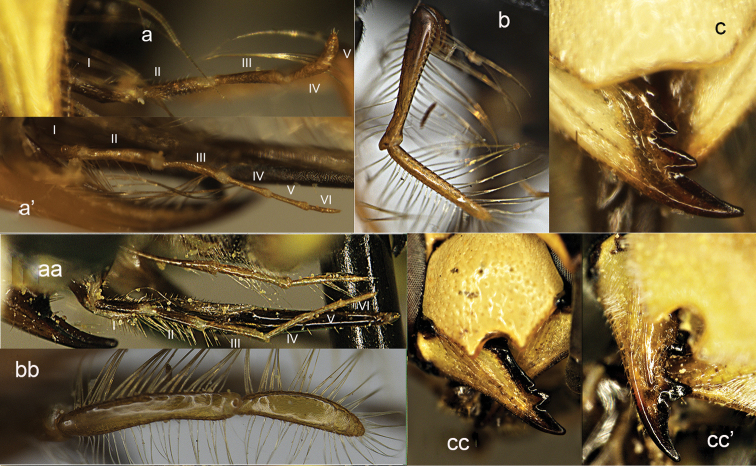
Maxillary palpus (a, a’ and aa), labial palpus of female (**b, bb**) and mandibles of male (**c, cc, cc**’). **a–c** *Pterocheilus p. phaleratus* (Panzer) **a**’ *Pterocheilus c. chobauti* Dusmet; **aa–bb** *Onychopterocheilus mochii* Giordani Soika **cc** *Onychopterocheilus pallasii* (Klug) **cc**’ *Onychopterocheilus* sp.

**Figure 21.**
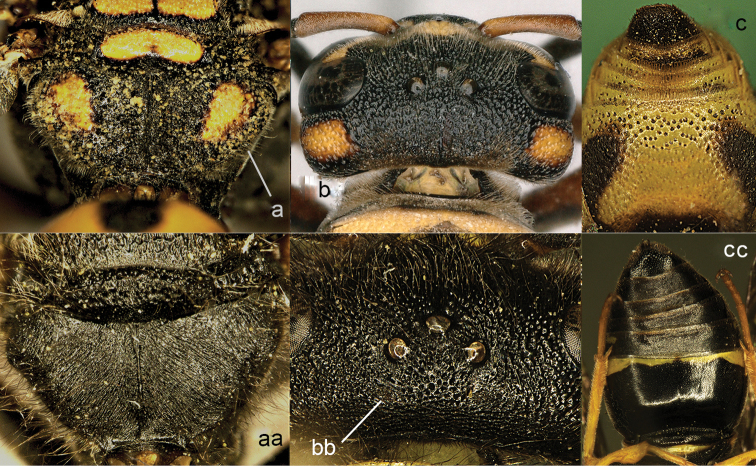
Propodeum (**a, aa**), head in dorsal view (**b, bb**) and sterna (**c, cc**). **a, b** *Tropidodynerus f. flavus* (Bingham) **c** *Tropidodynerus hostis* (Nurse) **aa–cc** *Odynerus albopictus* (de Saussure).

**Figure 22.**
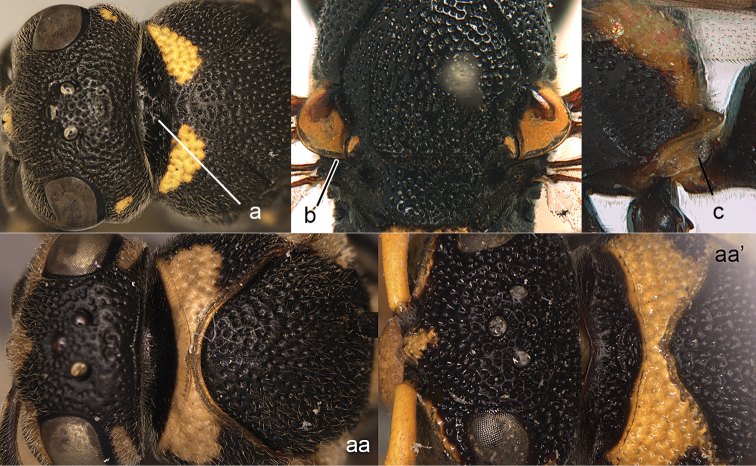
Head and pronotum in dorsal view (**a, aa, aa**’), part of mesosoma in dorsal view (**b**) and propodeum lateral view (**c**). **a, c** *Stenodynerus c. chinensis* (de Saussure) **b** *Stenodynerus frauenfeldi* (de Saussure) **aa** *Stenodyneriellus* sp. **aa**’ *Brachyodynerus magnificus* (Morawitz).

**Figure 23.**
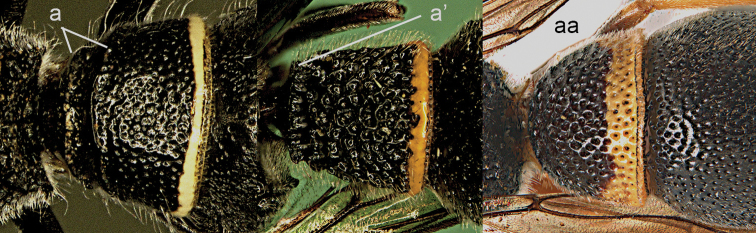
Metasomal tergum I. **a** *Subancistrocerus sichelii* (de Saussure) **a**’ *Pseudonortonia abbreviaticornis* Giordani Soika **aa** *Stenodynerus chinensis* (de Saussure).

**Figure 24.**
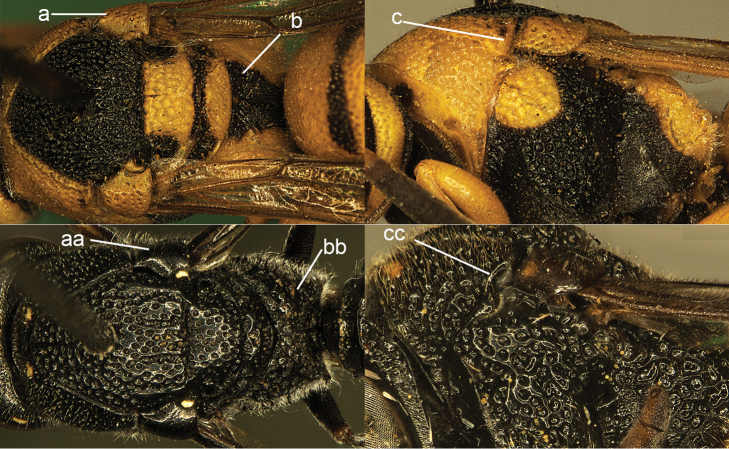
Mesosoma in dorsal view (**a, b, aa, bb**) and in lateral view (**c, cc**). **a–c** Jucancistrocerus (Eremodynerus) atrofasciatus (Morawitz) **aa–cc** *Pseudonortonia abbreviaticornis* Giordani Soika.

**Figure 25.**
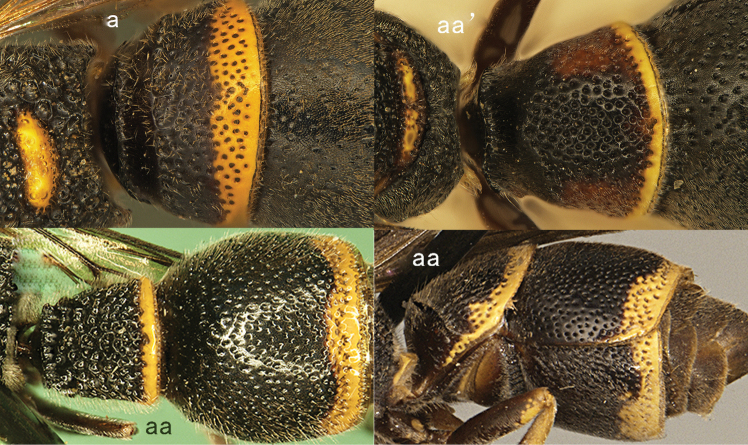
Metasomal tergum I in dorsal view. **a** *Subancistrocerus domesticus* **aa** (left). *Pseudonortonia abbreviaticornis* Giordani Soika **aa** (right). *Parancistrocerus samarensis* (von Schulthess) **aa**’ *Pseudonortonia* sp.

**Figure 26.**
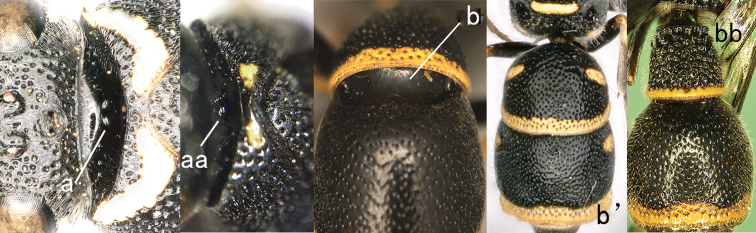
Anterior face of pronotum (**a, aa**); metasomal segments I and II (**b, b’, bb**). **a, b**’ *Parancistrocerus toltecus* **b** *Parancistrocerus samarensis* (von Schulthess) **bb** *Pseudonortonia abbreviaticornis* Giordani Soika.

**Figure 27.**
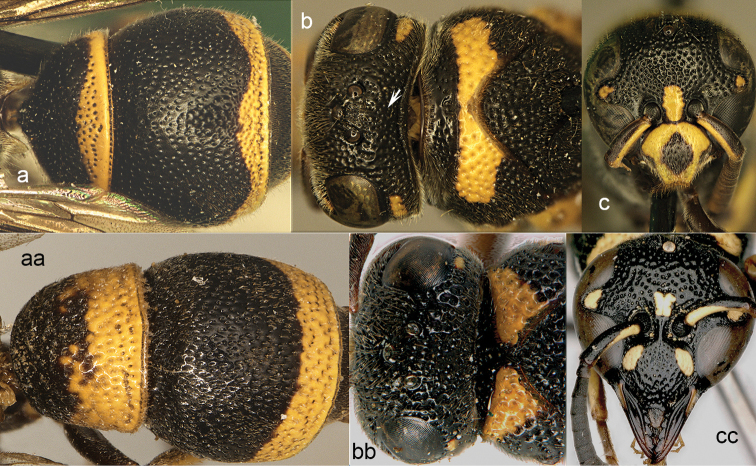
Metasoma in dorsal view (**a, aa**), head and pronotum in dorsal view (**b, bb**, white-arrow pointing to the depression) and in frontal view (**c, cc**). *Paraleptomenes kosempoensis* (von Schulthess) **aa–cc** *Stenodynerus chinensis* (de Saussure).

**Figure 28.**
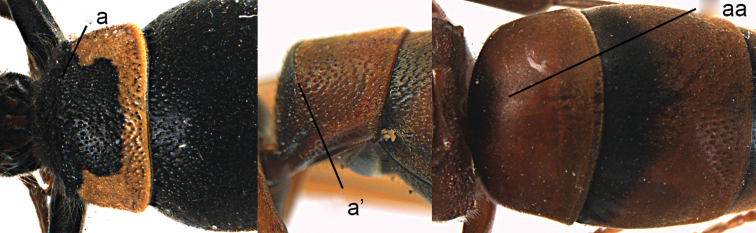
Metasomal tergum I (TI) in dorsal view (**a, aa**) and in lateral view (**a**’). **a** *Ancistrocerus parietinus* (Linnaeus) **a**’ *Pararrhynchium ornatum sauteri* (Schulthess) **c** *Rhynchium carnaticum* (Fabricius).

**Figure 29.**
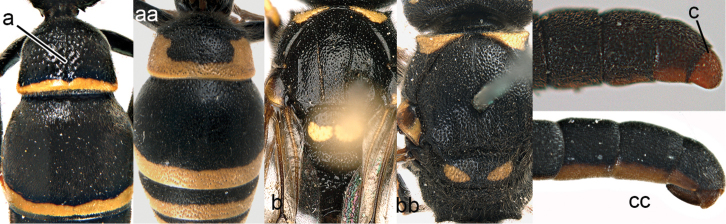
Metasomal terga I-II (**a, aa**) and mesosoma (**b, bb**) in dorsal view, distal segments of antenna (**c, cc**). **a, c** *Symmorphus bifasciatus* (Linnaeus) **b** *Symmorphus elegans* **aa–cc** *Ancistrocerus parietinus* (Linnaeus).

**Figure 30.**
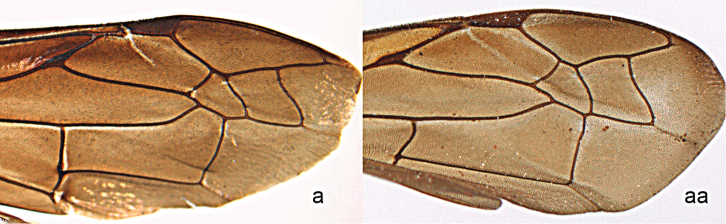
Forewing. **a** *Orancistrocerus a. aterrimus* (de Saussure) **aa** *Pararrhynchium o. ornatum* (Smith).

**Figure 31.**
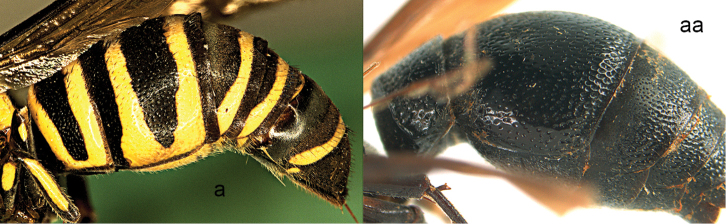
Metasoma in lateral view. **a** *Lissodynerus s. septemfasciatus* (Smith) **aa** *Orancistrocerus a. aterrimus* (de Saussure).

**Figure 32.**
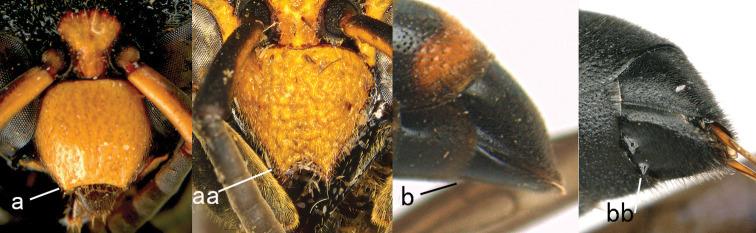
Clypeus (2 left) and distal part of male metasoma in lateral view (2 right). **a, b** *Orancistrocerus drewseni opulentissimus* (Giordani Soika) **aa** *Archancistrocerus diffinis* Giordani Soika, holotype **bb** *Archancistrocerus* sp.

**Figure 33.**
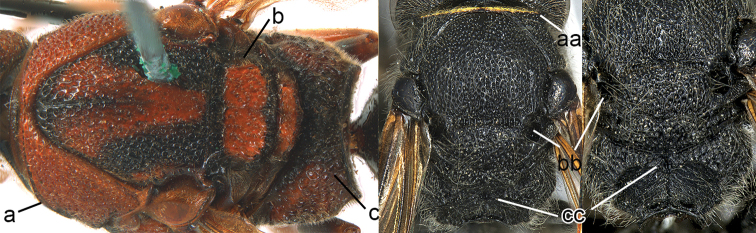
Mesosoma in dorsal view (left and middle) and dorso-caudal view (right). **a–c** *Pararrhynchium ornatum sauteri* (Schulthess) **aa–cc** *Ancistrocerus trifasciatus shibuyai* (Yasumatsu).

**Figure 34.**
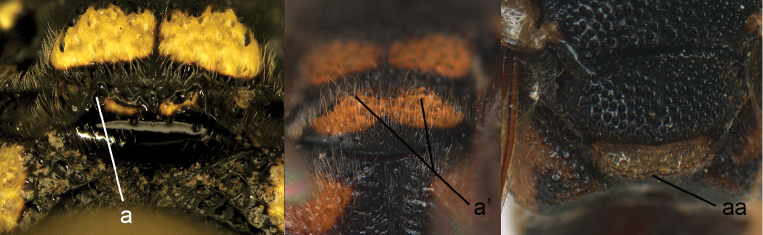
Metanotum. a. *Antepipona asiamontana* Gusenleitner; **a**’ *Apodynerus f. formosensis* (von Schulthess) **aa** *Euodynerus trilobus* (Fabricius).

**Figure 35.**
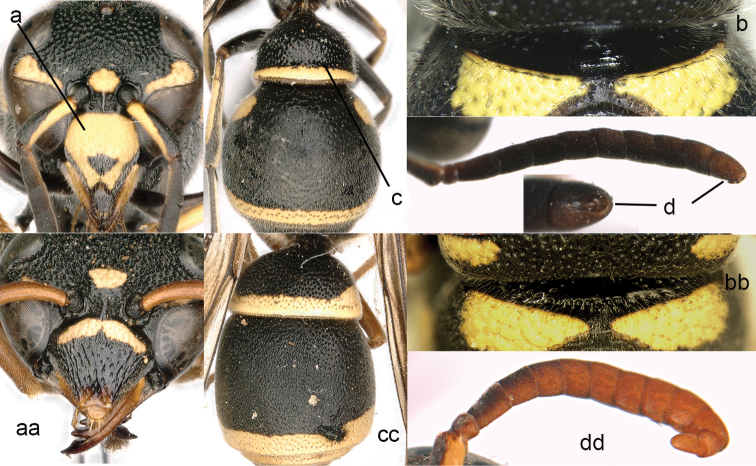
Head in frontal view (**a, aa**), metasoma in dorsal view (**c, cc**), anterior face of pronotum (**b, bb**) and antenna (**d, dd**). **a–d** *Apodynerus troglodytes* (de Saussure) **aa, cc** *Antepipona silaos* (de Saussure) **bb** *Antepipona menkei* Giordani Soika; dd. *Antepipona rufescens* (Smith).

**Figure 36.**
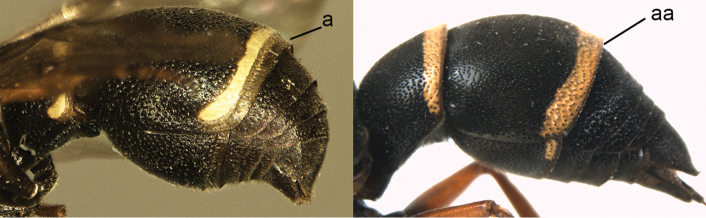
Metasoma in lateral view. **a** *Leptochilus m. medane* (Gribodo) **aa** *Anterhynchium flavomarginatum micado* (Kirsch).

**Figure 37.**
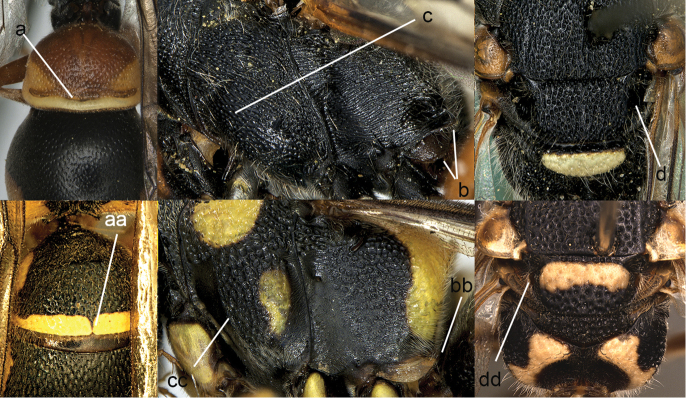
Metasomal tergum I (**a, aa**) in dorsal view, propodeum in lateral view (**b, bb**), mesosoma in lateral view (**c, cc**) and metanotum in dorsal view (**d, dd**). **a–d** *Leptochilus m. mauritanicus* (Lepeletier) **aa** *Gribodia confluenta* (Smith) **bb, cc** *Stenodyneriellus guttulatus* (de Saussure) **dd** *Stenodyneriellus* sp.

**Figure 38.**
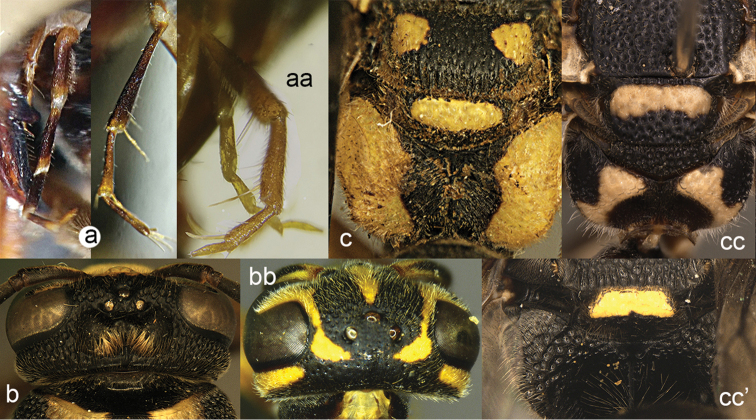
Maxillary palpus (**a** left) and labial palpus (**b** right), mouthpart palpi (**aa**), head in dorsal view (**b, bb**), part of mesosoma in dorso-caudal view (**c, cc, cc**’). **a–c** *Gribodia* sp. **aa, bb** *Stenodyneriellus guttulatus* (de Saussure) **cc** *Stenodyneriellus* sp. **cc**’ *Epsilon dyscherum* (de Saussure).

**Figure 39.**
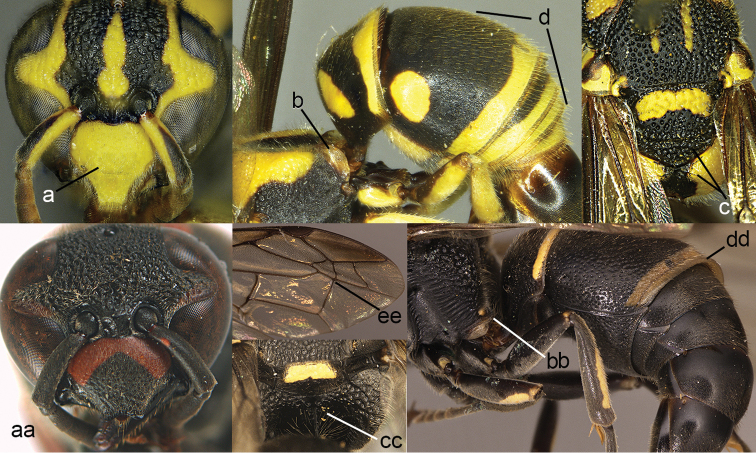
Head in frontal view (**a, aa**), part of meso- and metasoma (**b, d, bb, dd**), part of mesosoma in dorsal view (**c, cc**) and distal part of forewing (**ee**). **a–d** *Stenodyneriellus guttulatus* (de Saussure) **aa–ee** *Epsilon dyscherum* (de Saussure).

**Figure 40.**
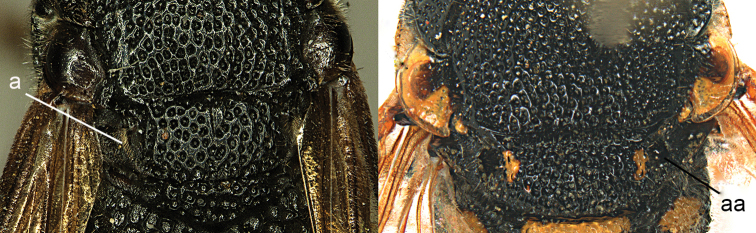
Part of mesosoma in dorsal view. **a** *Orientalicesa unifasciata* (von Schulthess) **aa** *Euodynerus p. posticus* (Herrich-Schäffer).

**Figure 41.**
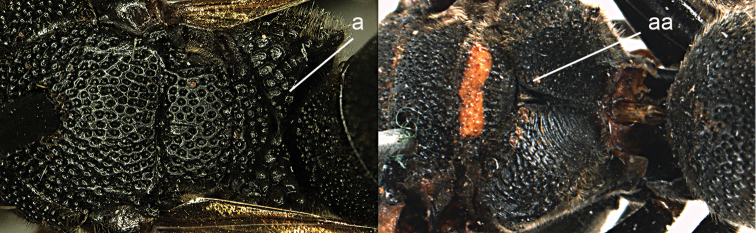
Part of mesosoma in dorsal view (**a**) and in dorso-caudal view (**aa**). **a** *Orientalicesa unifasciata* (von Schulthess) **aa** Anterhynchium (Dirhynchium) flavopunctatum (Smith).

**Figure 42.**
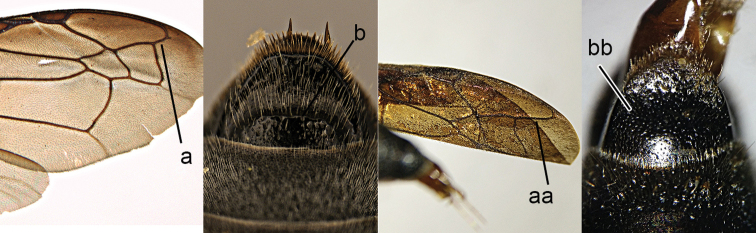
Distal part of forewing (**a, aa**) and terminal sternum of male (sternum VII) (**b, bb**). **a, b** *Allorhynchium argentatum* (Fabricius) **aa, bb** *Orientalicesa unifasciata* (von Schulthess).

**Figure 43.**
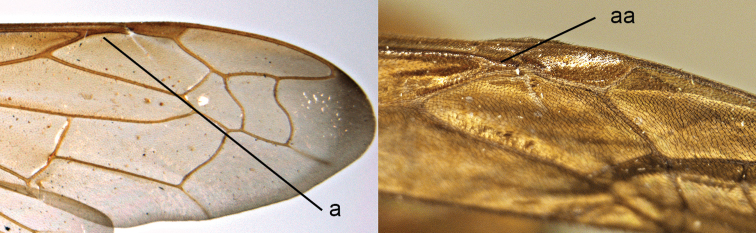
Part of forewing. **a** *Rhynchium carnaticum* (Fabricius) **b** *Okinawepipona kojimai* (Giordani Soika).

**Figure 44.**
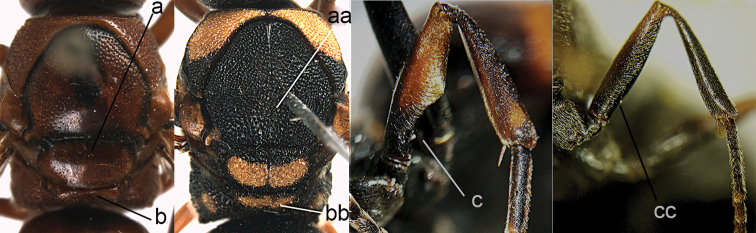
Mesosoma in dorsal view (**a, b, aa, bb**) and middle leg of male (**c, cc**). **a, b** *Rhynchium carnaticum* (Fabricius) **c** *Rhynchium q. quinquecinctum* (Fabricius) **aa, bb** *Anterhynchium flavomarginatum micado* (Kirsch) **cc** *Anterhynchium* sp.

**Figure 45.**
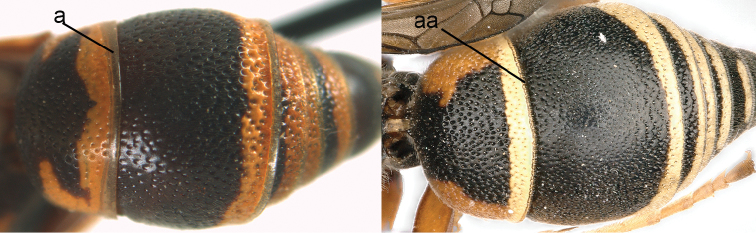
Metasoma in dorsal view. **a** *Euodynerus trilobus* (Fabricius) **aa** *Pseudepipona h. herrichii* (de Saussure).

**Figure 46.**
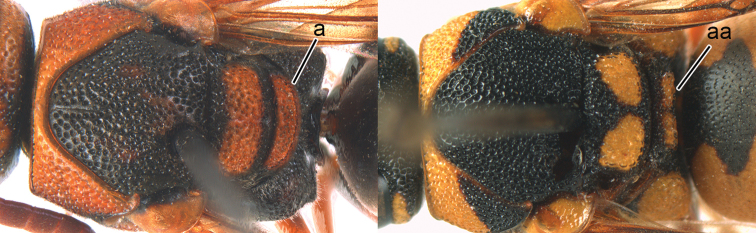
Mesosoma in dorsal view. **a** *Antodynerus limbatus* (de Saussure) **aa** *Euodynerus d. dantici* (Rossi).

**Figure 47.**
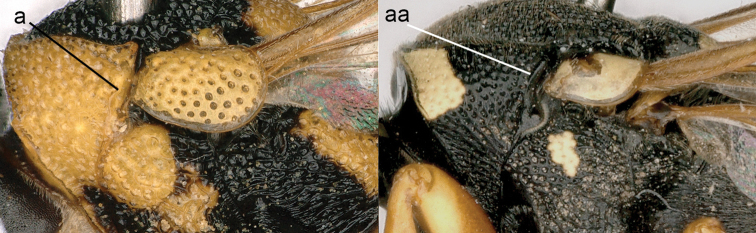
Part of mesosoma in lateral view. **a** *Brachyodynerus m. magnificus* (Morawitz) **aa** *Pseudepipona h. herrichii* (de Saussure).

**Figure 48.**
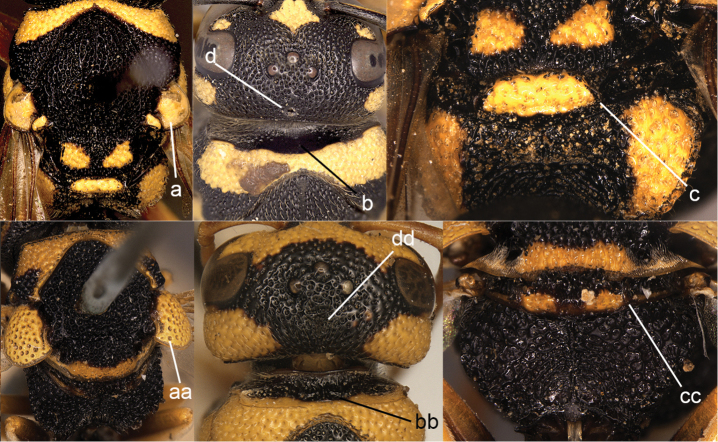
Mesosoma in dorsal view (**a, aa**), head and pronotum in dorsal view (**b, d, bb, dd**) and metanotum in dorsal view (**c, cc**). **a–d** *Parodontodynerus e. ephippium* (Klug) **aa–dd** *Brachyodynerus magnificus* (Morawitz).

**Figure 49.**
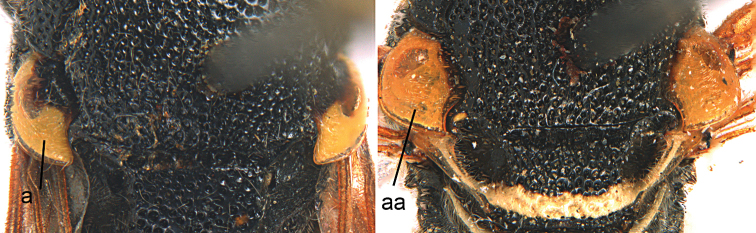
Part of mesonotum showing tegula and parategula. **a** *Allodynerus mandschuricus* Blüthgen **aa** *Pseudepipona herrichii siberia* Kurzenko.

**Figure 50.**
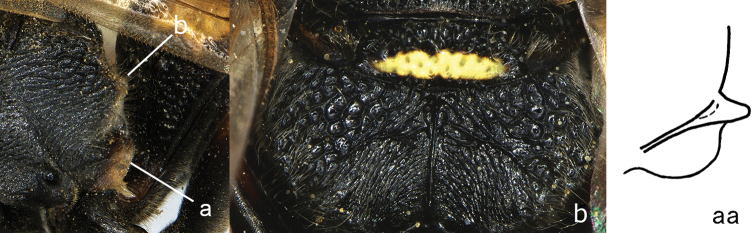
Propodeum in lateral view (**a, aa**) and in caudal view (**b**). **a, b** *Pseudepipona herrichii siberia* Kurzenko **aa** *Asiodynerus lucifer* (Kostylev), after Kurzenko (1977).

### Checklist of the species of the subfamily Eumeninae from China

***Allodynerus* Blüthgen, 1938**

*Allodynerus* Blüthgen, 1938 (1937), Konowia 16: 280; Type species: “*Lionotus floricola* Sauss. 1852” [= *Odynerus floricola* de Saussure, 1853].

*Allodynerus delphinalis delphinalis* (Giraud, 1866)

*Allodynerus mandschuricus* Blüthgen, 1953

***Allorhynchium* van der Vecht, 1963**

*Allorhynchium* van der Vecht, 1963, Zool. Verh., Leiden 60: 57 (key), 58, genus. Type species: *Vespa argentata* Fabricius, 1804.

*Allorhynchium chinense* (de Saussure, 1862)

*Allorhynchium lugubrinum* (Cameron, 1900)

*Allorhynchium metallicum* (de Saussure, 1852)

***Ancistrocerus* Wesmael, 1836**

*Ancistrocerus* Wesmael, 1836, Bull. Acad. R. Belg. 3: 45. Type species: *Vespa parietum* Linnaeus, 1758.

*Ancistrocerus arcanus* Giordani Soika, 1993

*Ancistrocerus aureovillosus* Giordani Soika, 1977

*Ancistrocerus deqinensis* You and Li, 2013

*Ancistrocerus frigidus* Giordani Soika, 1977 (1976)

*Ancistrocerus hirsutus hirsutus* (Meade-Waldo, 1910)

*Ancistrocerus hirsutus supiensis* Giordani Soika, 1977 (1976)

*Ancistrocerus hirsutus tinkiensis* Giordani Soika, 1977 (1976)

*Ancistrocerus khangmarensis* Giordani Soika, 1966

*Ancistrocerus krausei* Giordani Soika, 1966

*Ancistrocerus melanocerus* (Dalla Torre, 1894)

*Ancistrocerus melanurus* Morawitz, 1889

*Ancistrocerus montuosus* Gusenleitner, 1993

*Ancistrocerus nigricornis* (Curtis, 1791)

*Ancistrocerus parapoloi* Giordani Soika, 1966

*Ancistrocerus parietum* (Linnaeus, 1758)

*Ancistrocerus rufofrustius* Tan & Carpenter, **nom. n.**

Replacement name for *Ancistrocerus rufopictus* (Kostylev, 1940) (junior primary homonym of Odynerus lineaticollis var. rufopictus Meade-Waldo, 1915).

*Ancistrocerus sikhimensis* (Bingham, 1897)

*Ancistrocerus terayamai* Yamane, 1993

*Ancistrocerus tibetanus* Giordani Soika, 1966

*Ancistrocerus transpunctatus* You & Li, 2013

*Ancistrocerus trifasciatus shibuyai* (Yasumatsu, 1938)

*Ancistrocerus waltoni* (Meade-Waldo, 1910)

***Antepipona* de Saussure, 1855**

*Antepipona* de Saussure, 1855, Ét. Fam. Vesp. 3: 244. Type species: *Odynerus silaos* de Saussure, 1853.

*Antepipona asiamontana* Gusenleitner, 2004

*Antepipona biguttata* (Fabricius, 1787)

*Antepipona bipustulata* (de Saussure, 1855)

*Antepipona brunneola* Giordani Soika, 1986

*Antepipona deflenda* (Saunders, 1853)

*Antepipona excelsa excelsa* Giordani Soika, 1982

*Antepipona ferruginea* Kim & Yamane, 2003

*Antepipona ferruginea* Kim & Yamane, 2003

*Antepipona guttata diffinis* (de Saussure, 1855)

*Antepipona menkei* Giordani Soika, 1986

*Antepipona ovalis* (de Saussure, 1853)

*Antepipona plurimacula* Giordani Soika, 1971

*Antepipona rufescens* (Smith, 1857)

*Antepipona shantungensis* Giordani Soika, 1993

*Antepipona sexfasciata* Soika, 1986

*Antepipona tytides* (Cameron, 1904)

***Anterhynchium* de Saussure, 1863**

*Anterhynchium* de Saussure, 1863, Mém. Soc. Phys. Hist. Nat. Genève 17: 205. Type species: *Rygchium* [= *Rhynchium*] *synagroides* de Saussure, 1852.

Anterhynchium (Anterhynchium) mellyi (de Saussure, 1852)

Anterhynchium (Dirhynchium) flavomarginatum flavomarginatum (Smith, 1852)

Anterhynchium (Dirhynchium) flavomarginatum formosicola (von Schulthess, 1934)

Anterhynchium (Dirhynchium) flavopunctatum flavopunctatum (Smith, 1852)

Anterhynchium (Dirhynchium) flavopunctatum opulentum (Giordani Soika, 1973)

Anterhynchium (Dirhynchium) inamurai (Sonan, 1937)

Anterhynchium (Dirhynchium) yunnanensis Giordani Soika, 1973

***Antodynerus* de Saussure, 1855**

*Antodynerus* de Saussure, 1855, Ét. Fam. Vesp. 3: 287.Type species: “*Odynerus punctum* (Fabricius)” sensu de Saussure, 1853 [= *Vespa flavescens* Fabricius, 1775]

*Antodynerus limbatus* (de Saussure, 1852)

***Apodynerus* Giordani Soika, 1993**

*Apodynerus* Giordani Soika, 1993, Boll. Mus. Civ. Stor. Nat. Venezia 42: 155. Type species: *Odynerus troglodytes* de Saussure, 1855.

*Apodynerus formosensis continentalis* Giordani Soika, 1994

*Apodynerus formosensis formosensis* (von Schulthess, 1934)

*Apodynerus troglodytes troglodytes* (de Saussure, 1855)

*Apodynerus yayeyamensis yayeyamensis* (Matsmura, 1926)

***Archancistrocerus* Giordani Soika, 1986**

*Archancistrocerus* Giordani Soika, 1986, Boll. Mus. Civ. Stor. Nat. Venezia 35: 143, genus. Type species: *Archancistrocerus diffinis* Giordani Soika, 1986, monotypy.

*Archancistrocerus diffinis* Giordani Soika, 1986

Note: *Archancistrocerus* is a junior synonym and will be included in *Allorhynchium* (Tan et al. 2018b, submitted).

***Asiodynerus* Kurzenko, 1977**

*Asiodynerus* Kurzenko, 1977, Ins. Mongol. 5: 557. Type species: *Odynerus lucifer* Kostylev, 1937.

*Asiodynerus lucifer* (Kostylev, 1937 (1936))

***Brachyodynerus* Blüthgen, 1938**

*Brachyodynerus* Blüthgen, 1938, Deutsch. Entomol. Zeitschr.: 450, 459, genus. Type species: Odynerus (Lionotus) magnificus Morawitz, 1867.

*Brachyodynerus perarrus* Kurzenko, 1977

***Calligaster* de Saussure, 1852**

*Calligaster* de Saussure, 1852, Ét. fam. Vesp. 1: 22, genus. Type species: *Calligaster cyanoptera* de Saussure, 1852.

*Calligaster himalayensis* (Cameron, 1904)

***Coeleumenes* van der Vecht, 1963**

*Coeleumenes* van der Vecht, 1963, Zool. Verh., Leiden 60: 45, genus. Type species: *Montezumia impavida* Bingham, 1897

*Coeleumenes burmanicus* (Bingham, 1897)

*Coeleumenes thoracicus* (Sonan, 1939)

***Cyrtolabulus* van der Vecht, 1969**

*Cyrtolabulus* van der Vecht, 1969, Entomol. Ber., Amst. 29: 1, replacement name for *Cyrtolabus* van der Vecht, 1963, *non* Voss, 1925. Type species: *Cyrtolabus suavis* van der Vecht, 1963.

*Cyrtolabulus exiguus* (de Saussure, 1853)

*Cyrtolabulus yunnanensis* Lee, 1982

***Delta* de Saussure, 1855**

*Delta* de Saussure, 1855, Ét. Fam. Vesp. 3: 130, 132, 143. Type species: *Vespa maxillosa* DeGeer, 1775 [= *Vespa emarginata* Linnaeus, 1758]

*Delta campaniforme campaniforme* (Fabricius, 1775)

*Delta conoideum* (Gmelin, 1790)

*Delta esuriens okinawae* Giordani Soika, 1986

*Delta pyriforme pyriforme* (Fabricius, 1775)

***Discoelius* Latreille, 1809**

*Discoelius* Latreille, 1809, Gen. Crust. et Insect. 4: 140. Type species: *Vespa zonalis* Panzer, 1801, monotypy.

*Discoelius dufourii dufourii* (Lepeletier, 1841)

*Discoelius dufourii manchurianus* Yasumatsu, 1934

*Discoelius emeishanensus* Zhou and Li, 2013

*Discoelius esakii* Yasumatsu, 1934

*Discoelius longinodus* Yamane, 1996

*Discoelius nigriclypeus* Zhou & Li, 2013

*Discoelius wangi* Yamane, 1996

*Discoelius zonalis* (Panzer, 1801)

***Ectopioglossa* Perkins, 1912**

*Ectopioglossa* Perkins, 1912, Ann. Mag. Nat. Hist. (8) 9: 118, genus. Type species: *Ectopioglossa australensis* Perkins, 1912, monotypy.

*Ectopioglossa ovalis* Giordani Soika, 1993

*Ectopioglossa taiwana* (Sonan, 1938)

***Epsilon* Saussure, 1855**

*Epsilon* de Saussure, 1855, Ét. Fam. Vesp. 3: 229, 252. Type species: *Odynerus dyscherus* de Saussure, 1852.

*Epsilon fujianense* Lee, 1981

***Eumenes* Latreille, 1802**

*Eumenes* Latreille, 1802, Hist. Nat. Crust. Ins. 3: 360, genus. Type species: “*Eumenes coarctata*, Fab.” [= *Vespa coarctata* Linnaeus, 1758].

*Eumenes architectus* Smith, 1859

*Eumenes assamensis* Meade-Waldo, 1910

*Eumenes atrophicus* (Fabricius, 1798)

*Eumenes buddha* Cameron, 1897

*Eumenes coarctatus coarctatus* (Linnaeus, 1758)

*Eumenes coronatus coronatus* (Panzer, 1799)

*Eumenes ferrugiantennus* Zhou, Chen & Li, 2012

*Eumenes formosensis* Giordani Soika, 1973

*Eumenes fraterculus* Dalla Torre, 1894

*Eumenes fulvopilosellus* Giordani Soika, 1965

*Eumenes kangrae* Dover, 1925

*Eumenes kiangsuensis* Giordani Soika, 1941

*Eumenes labiatus flavoniger* Giordani Soika, 1941

*Eumenes labiatus labiatus* Giordani Soika, 1941

*Eumenes labiatus sinicus* Giordani Soika, 1941

*Eumenes mediterraneus manchurianus* Giordani Soika, 1971

*Eumenes micado* Cameron, 1904 (Kim and Yoon (2001) state that the record is very doubtful)

*Eumenes multipictus* de Saussure, 1855

*Eumenes nigriscutatus* Zhou, Chen & Li, 2012

*Eumenes pedunculatus pedunculatus* (Panzer, 1799)

*Eumenes pomiformis* (Fabricius, 1781)

*Eumenes punctatus* de Saussure, 1852

*Eumenes quadratus obsoletus* Dover, 1926

*Eumenes quadratus quadratus* Smith, 1852

*Eumenes quadratus urainus* Sonan, 1939

*Eumenes rubronotatus* Pérez, 1905

*Eumenes septentrionalis khangmarensis* Giordani Soika, 1966

*Eumenes septentrionalis septentrionalis* Giordani Soika, 1940

*Eumenes tosawae lofouensis* Giordani Soika, 1973

*Eumenes tosawae tosawae* Giordani Soika, 1934

*Eumenes transbaicalicus* Kurzenko, 1984

*Eumenes tripunctatus* (Christ, 1791)

*Eumenes variepunctatus* Giordani Soika, 1941

***Euodynerus* Dalla Torre, 1904**

*Euodynerus* Dalla Torre, 1904, Gen. Ins. 19: 38. Type species: *Vespa dantici* Rossi, 1790.

Euodynerus (Euodynerus) caspicus caspicus (Morawitz, 1873)

Euodynerus (Euodynerus) dantici brachytomus (Kostylev, 1940)

Euodynerus (Euodynerus) dantici violaceipennis Giordani Soika, 1973

Euodynerus (Euodynerus) fastidiosus (de Saussure, 1853)

Euodynerus (Euodynerus) rufinus rufinus Blüthgen, 1942

Euodynerus (Euodynerus) semisaecularis semisaecularis (Dalla Torre, 1889)

Euodynerus (Euodynerus) variegatus kruegeri (von Schulthess, 1928)

Euodynerus (Pareuodynerus) adiacens Giordani Soika, 1973

Euodynerus (Pareuodynerus) deqinensis Ma, Chen & Li, 2017

Euodynerus (Pareuodynerus) ferrugineus Ma, Chen & Li, 2017

Euodynerus (Pareuodynerus) nipanicus nipanicus (von Schulthess, 1908)

Euodynerus (Pareuodynerus) nipanicus ryukyuensis Tano, 1987

Euodynerus (Pareuodynerus) nipanicus tonkinensis Giordani Soika, 1973

Euodynerus (Pareuodynerus) notatus notatus (Jurine, 1807)

Euodynerus (Pareuodynerus) quadrifasciatus quadrifasciatus (Fabricius, 1793)

Euodynerus (Pareuodynerus) similinipanicus Ma, Chen & Li, 2017

Euodynerus (Pareuodynerus) strigatus (Radoszkowski, 1893)

Euodynerus (Pareuodynerus) trilobus (Fabricius, 1787)

***Gribodia* Zavattari, 1912**

*Gribodia* Zavattari, 1912, Arch. Naturgesch. 78A (4): 161. Type species: *Monobia cavifrons* Gribodo, 1891 [= *Odynerus confluenta* Smith, 1857], monotypy.

*Gribodia nigra* Nguyen & Xu, 2015

***Jucancistrocerus* Blüthgen, 1938**

*Jucancistrocerus* Blüthgen, 1938, Deutsch. Entomol. Zeitschr.: 442, 460.Type species: Odynerus (Ancistrocerus) jucundus Mocsáry, 1883, monotypy.

Jucancistrocerus (Jucancistrocerus) alashanicus Kurzenko, 1977

Jucancistrocerus (Jucancistrocerus) angustifrons (Kostylev, 1940)

Jucancistrocerus (Eremodynerus) atrofasciatus (Morawitz, 1885)

Jucancistrocerus (Eremodynerus) chotanensis (Blüthgen, 1942)

***Katamenes* Meade-Waldo, 1910**

*Katamenes* Meade-Waldo, 1910, Ann. Mag. Nat. Hist. (8) 5: 46, genus. Type species: *Katamenes watsoni* Meade-Waldo, 1910, monotypy.

*Katamene arbustorum arbustorum* (Panzer, 1799)

*Katamene indetonsus* (Morawitz, 1895)

*Katamene tauricus tauricus* (de Saussure, 1855)

***Labus* de Saussure, 1867**

*Labus* de Saussure, 1867, Reise Novara, Zool. 2 (1), Hym.: 3, genus. Type species: *Labus spiniger* de Saussure, 1867

*Labus exiguus* (de Saussure, 1855)

*Labus lofuensis* Giordani Soika, 1973

***Leptochilus* de Saussure, 1853**

*Leptochilus* de Saussure, 1853, Ét. Fam. Vesp 1: 233. Type species: *Pterocheilus mauritianus* [!] [= *Pterocheilus mauritanicus* Lepeletier, 1841].

Leptochilus (Lionotulus) chinensis Gusenleitner, 2001

Leptochilus (Lionotulus) gobicus (Kostylev, 1940)

Leptochilus (Lionotulus) incertus (Kostylev, 1940)

Leptochilus (Neoleptochilus) tibetanus Giordani Soika, 1966

***Lissodynerus* Giordani Soika, 1993**

*Lissodynerus* Giordani Soika, 1993, Boll. Mus. Civ. Stor. Nat. Venezia 42: 135. Type species: *Odynerus septemfasciatus* Smith, 1857.

*Lissodynerus septemfasciatus feanus* (Giordani Soika, 1941)

***Leptomicrodynerus* Giordani Soika, 1985**

*Leptomicrodynerus* Giordani Soika, 1985, Lavori Soc. Ven. Sci. Nat. 10: 37. Type species: *Leptomicrodynerus tieshengi* Soika, 1985, monotypy.

*Leptomicrodynerus tieshengi* Giordani Soika, 1985

Note: The characteristics of this genus also fit well with *Eumenidiopsis* Giordani Soika, 1939, and further research is needed to solve the problem.

***Nortozumia* van der Vecht, 1937 (new record)**

*Nortozumia* van der Vecht, 1937, Treubia 16: 263, genus. Type species: *Zethus rufofemoratus* Cameron, 1903.

*Nortozumia* sp.

***Odynerus* Latreille, 1802**

*Odynerus* Latreille, 1802, Hist. Nat. Crust. Ins. 3: 362. Type species: *Vespa spinipes* Linnaeus, 1758.

Odynerus (Odynerus) tristis tianshanicus Kurzenko, 1977.

***Okinawepipona* Yamane, 1987**

*Okinawepipona* Yamane, 1987, Mem. Kagoshima Univ. Res. Center S. Pacific 8: 52. Type species: *Anterhynchium kogimai* Giordani Soika, 1986, monotypy.

*Okinawepipona curcipunctura* Nguyen & Xu, 2014

*Okinawepipona kogimai taiwana* Yamane, 1987

*Okinawepipona nigra* Nguyen & Xu, 2014

***Onychopterocheilus* Blüthgen, 1955**

*Onychopterocheilus* Blüthgen, 1955, Mitt. Münch. Entomol. Ges. 44/45: 406, 407. Type species: “*Pterocheilus daw* (Dusmet, 1909)” [= Odynerus (Hoplomerus) daw Dusmet, 1903], monotypy.

Onychopterocheilus (Asiapterocheilus) bensoni (Giordani Soika, 1941)

Onychopterocheilus (Asiapterocheilus) nigropilosus (Kostylev, 1940)

Onychopterocheilus (Asiapterocheilus) rongsharensis (Giordani Soika, 1977)

Onychopterocheilus (Asiapterocheilus) tibetanus (Meade-Waldo, 1913)

Onychopterocheilus (Asiapterocheilus) waltoni (Meade-Waldo, 1913)

Onychopterocheilus (Onychopterocheilus) chinensis Gusenleitner, 2005

Onychopterocheilus (Onychopterocheilus) dementievi (Kostylev, 1940)

Onychopterocheilus (Onychopterocheilus) eckloni (Morawitz, 1885)

Onychopterocheilus (Onychopterocheilus) wuhaiensis Gusenleitner, 2005

***Orancistrocerus* van der Vecht, 1963**

*Orancistrocerus* van der Vecht, 1963, Zool. Verh., Leiden 60: 58 (key), 99, genus. Type species: *Odynerus drewseni* de Saussure, 1857

*Orancistrocerus aterrimus aterrimus* (de Saussure, 1852)

*Orancistrocerus drewseni drewseni* (de Saussure, 1857)

*Orancistrocerus drewseni ingens* (von Schulthess, 1934)

*Orancistrocerus drewseni opulentissimus* (Giordani Soika, 1941)

*Orancistrocerus moelleri aulicus* Giordani Soika, 1973

***Oreumenes* Bequaert, 1926**

*Oreumenes* Bequaert, 1926, Ann. S. Afr. Mus. 23: 488. Type species: *Eumenes harmandi* Perez, 1905 [= *Eumenes decoratus* Smith, 1852], monotypy.

*Oreumenes decoratus* (Smith, 1852)

***Orientalicesa* Koçak & Kemal, 2010**

*Orientalicesa* Koçak & Kemal, 2010, CESA Misc. Pap. 151: 4, replacement name for *Kennethia* Giordani Soika, 1994, non De Dekker, 1979. Type species: *Odynerus unifasciatus* von Schulthess, 1934.

*Orientalicesa confasciatus* Tan & Carpenter, nom. n.

Replacement name for *Orientalicesa unifasciatus* (von Schulthess, 1934) (junior primary homonym of *Odynerus unifasciatus* de Saussure, 1852).

***Paraleptomenes* Giordani Soika, 1970**

*Paraleptomenes* Giordani Soika, 1970, Boll. Mus. Civ. Stor. Nat. Venezia 20/21: 79, genus. Type species: *Paraleptomenes nurseanus* Giordani Soika, 1970, monotypy.

*Paraleptomenes kosempoensis* (von Schulthess, 1934)

*Paraleptomenes miniatus miniatus* (de Saussure, 1855)

***Parancistrocerus* Bequaert, 1925**

*Parancistrocerus* Bequaert, 1925, Trans. Am. Entomol. Soc. 51: 64. Type species: *Odynerus fulvipes* de Saussure, 1855 [= *O. “flavipes* Fabricius” *sensu* de Saussure, 1852, *non Vespa flavipes* Fabricius, 1775].

*Parancistrocerus hongkongensis* Gusenleitner, 2002

*Parancistrocerus intermediatus* (Sonan, 1939)

*Parancistrocerus kuraruensis* (Sonan, 1939)

*Parancistrocerus nitobei* (Sonan, 1939)

*Parancistrocerus taihorinensis* (von Schulthess, 1934)

*Parancistrocerus taikonus* (Sonan, 1939)

*Parancistrocerus yachowensis konkunesis* Giordani Soika, 1994

*Parancistrocerus yachowensis yachowensis* Giordani Soika, 1986

*Parancistrocerus yamanei* Gusenleitner, 2000

***Pararrhynchium* de Saussure, 1855**

*Pararrhynchium* de Saussure, 1855, Ét. Fam. Vesp. 3: 173. Type species: *Rhynchium ornatum* Smith, 1852, monotypy.

*Pararrhynchium ornatum bifasciatulum* Giordani Soika, 1986

*Pararrhynchium ornatum infrenis* Giordani Soika, 1973

*Pararrhynchium ornatum multifasciatum* Giordani Soika, 1986

*Pararrhynchium ornatum ornatum* (Smith, 1852)

*Pararrhynchium ornatum sauteri* (von Schulthess, 1934)

*Pararrhynchium paradoxum paradoxum* (Gussakovskij, 1932)

*Pararrhynchium sinense* (von Schulthess, 1913)

*Pararrhynchium smithii* (de Saussure, 1855)

*Pararrhynchium taiwanum* Kim & Yamane, 2007

***Pareumenes* de Saussure, 1855**

*Pareumenes* de Saussure, 1855, Ét. Fam. Vesp. 3: 133. Type species: *Eumenes quadrispinosus* de Saussure, 1855.

Pareumenes (Nortonia) taiwanus (Sonan, 1937)

Pareumenes (Pareumenes) chinensis Liu, 1941

Pareumenes (Pareumenes) obtusus Liu, 1941

Pareumenes (Pareumenes) quadrispinosus acutus Liu, 1941

*Pareumenes quadrispinosus conjunctus* Liu, 1941

Pareumenes (Pareumenes) quadrispinosus interruptus Liu, 1941

Pareumenes (Pareumenes) quadrispinosus quadrispinosus (de Saussure, 1855)

Pareumenes (Pareumenes) quadrispinosus transitorus Liu, 1941

***Parodontodynerus* Blüthgen, 1938**

*Parodontodynerus* Blüthgen, 1938 (1937), Konowia 16: 280. Type species: *Eumenes ephippium* Klug, 1817.

*Parodontodynerus laudatus* (Kostylev, 1940)

***Phimenes* Giordani Soika, 1992**

*Phimenes* Giordani Soika, 1992, Lavori Soc. Ven. Sci. Nat. 17: 41, 66, genus, replacement name for *Phi* de Saussure, 1855, non de Saussure, 1854. Type species: *Vespa arcuata* Fabricius, 1775.

*Phimenes flavopictus flavopictus* (Blanchard, 1804)

*Phimenes flavopictus formosanus* (Zimmermann, 1931)

*Phimenes sparsipunctatus* Gusenleitner, 2002

***Pseudepipona* de Saussure, 1856**

*Pseudepipona* de Saussure, 1856, Ét. Fam. Vesp. 3: 309. Type species: *Odynerus herrichii* de Saussure, 1856, monotypy.

Pseudepipona (Pseudepipona) augusta (Morawitz, 1867)

Pseudepipona (Pseudepipona) herrichii herrichii (de Saussure, 1856)

Pseudepipona (Pseudepipona) lativentris rubricans Kurzenko, 1976

*Pseudepipona przewalskyi* (Morawitz, 1885)

***Pseudonortonia* Giordani Soika, 1936**

*Pseudonortonia* Giordani Soika, 1936, Ann. Mus. Civ. Stor. Nat. Genova 59: 268, genus. Type species: *Odynerus difformis* de Saussure, 1853

*Pseudonortonia abbreviaticornis* Giordani Soika, 1941

***Pseudozumia* de Saussure, 1875**

*Pseudozumia* de Saussure, 1875, Smithson. Misc. Coll. 254 (I): 128, division of genus *Montezumia* de Saussure. Type species: *Montezumia indica* de Saussure, 1855, monotypy.

*Pseudozumia indica indica* (de Saussure, 1855)

*Pseudozumia indica paulonotata* Giordani Soika, 1941

*Pseudozumia indosinensis* Giordani Soika, 1960

***Pseumenes* Giordani Soika, 1935**

*Pseumenes* Giordani Soika, 1935, Ann. Mus. Civ. Stor. Nat. Genova 57: 145. Type species: *Eumenes eximius* Smith, 1861.

*Pseumenes depressus depressus* (de Saussure, 1855)

*Pseumenes imperatrix* (Smith, 1857)

***Pterocheilus* Klug, 1805**

*Pterocheilus* Klug, 1805, Beitr. Naturk. 1: 143. Type species: *Vespa phalerata* Panzer, 1797.

*Pterocheilus albofasciatus* Smith, 1878

*Pterocheilus napalkovi* Kurzenko, 1977

***Rhynchium* Spinola, 1806**

*Rhynchium* Spinola, 1806, Ins. Ligur. 1: 84. Type species: *Rygchium europaeum* Spinola, 1806 [= *Vespa oculata* Fabricius, 1781], monotypy.

*Rhynchium brunneum brunneum* (Fabricius, 1793)

*Rhynchium quinquecinctum quinquecinctum* (Fabricius, 1787)

***Stenodyneriellus* Giordani Soika, 1962**

*Stenodyneriellus* Giordani Soika, 1962 (1961), Boll. Mus. Civ. Stor. Nat. Venezia 14: 65, 71. Type species: *Stenodyneriellus turneriellus* Giordani Soika, 1962.

*Stenodyneriellus depressus* Li & Chen, 2016

*Stenodyneriellus guttulatus* (de Saussure, 1862)

*Stenodyneriellus maolanensis* Li & Chen, 2016

*Stenodyneriellus similiguttulatus* Li & Chen, 2016

***Stenodynerus* de Saussure, 1863**

*Stenodynerus* de Saussure, 1863, Mém. Soc. Phys. Hist. Nat. Genève 17: 228. Type species: *Odynerus chinensis* de Saussure, 1863.

*Stenodynerus baronii* Giordani Soika, 1975

*Stenodynerus bluethgeni* van der Vecht, 1971

*Stenodynerus chinensis chinensis* (de Saussure, 1863)

*Stenodynerus clyppeopictus* (Kostylev, 1940)

*Stenodynerus copiosus* Gusenleitner, 2012

*Stenodynerus frauenfeldi* (de Saussure, 1867)

*Stenodynerus funebris* (André, 1884)

*Stenodynerus incurvitus* Gusenleitner, 2003

*Stenodynerus morawitzi* Kurzenko, 1977

*Stenodynerus morbillosus* Giordani Soika, 1979

*Stenodynerus nepalensis* Giordani Soika, 1985

*Stenodynerus ninglangensis* Ma & Li, 2016

*Stenodynerus nudus* (Morawitz, 1889)

*Stenodynerus pappi luteifasciatus* Kim & Yamane, 2004

*Stenodynerus pappi pappi* Giordani Soika, 1976

*Stenodynerus pullus* Gusenleitner, 1981

*Stenodynerus reflexus* Ma & Li, 2016

*Stenodynerus similibaronii* Ma & Li, 2016

*Stenodynerus taiwanus* Kim & Yamane, 2004

*Stenodynerus tenuilamellatus* Ma & Li, 2016

*Stenodynerus tergitus* Kim, 1999

***Subancistrocerus* de Saussure, 1855**

*Subancistrocerus* de Saussure, 1855, Ét. Fam. Vesp. 3: 206. Type species: *Odynerus sichelii* de Saussure, 1855.

*Subancistrocerus camicrus* (Cameron, 1904)

*Subancistrocerus compressus* Li & Chen, 2014

*Subancistrocerus jinghongensis* Li & Chen, 2014

*Subancistrocerus kankauensis* (von Schulthess, 1934)

*Subancistrocerus sichelii* (de Saussure, 1855)

***Symmorphus* Wesmael, 1836**

*Symmorphus* Wesmael, 1836, Bull. Acad. R. Belg. 3: 45. Type species: *Odynerus elegans* Wesmael, 1833.

*Symmorphus ambotretus* Cumming, 1989

*Symmorphus angustatus* (Zetterstedt, 1838)

*Symmorphus apiciornatus* (Cameron, 1911)

*Symmorphus aurantiopictus* Giordani Soika, 1986

*Symmorphus bifasciatus* (Linnaeus, 1761)

*Symmorphus cavatus* Li and Chen, 2014

*Symmorphus foveolatus* Gussakovskij, 1933

*Symmorphus fuscipes* (Herrich-Schaeffer, 1838)

*Symmorphus hoozanensis* (von Schulthess, 1934)

*Symmorphus lucens* (Kostylev, 1938)

*Symmorphus mizuhonis* Tsuneki, 1977

*Symmorphus nigriclypeus* Li & Chen, 2014

*Symmorphus ornatus* Gusenleitner, 2000

*Symmorphus sichuanensis* Lee, 1981

*Symmorphus sublaevis* (Kostylev, 1940)

*Symmorphus tianchiensis* Li & Chen, 2014

*Symmorphus violaceipennis* Giordani Soika, 1966

*Symmorphus yananensis* Gusenleitner, 2002

*Symmorphus yunnanensis* Gusenleitner, 2002

***Tropidodynerus* Blüthgen, 1939**

*Tropidodynerus* Blüthgen, 1939, Veröff. Deutsch. Kolon. Übersee Mus. Bremen 2: 259, 260. Type species: “*Hoplomerus interruptus* (Brullé, 1832) = *H. mandibularis* Morawitz, 1885” [= *Polistes interrupta* Brullé, 1832].

*Tropidodynerus concavus* Li & Chen, 2015

*Tropidodynerus liupanshanensis* Li & Chen, 2015

***Zethus* Fabricius, 1804**

*Zethus* Fabricius, 1804, Syst. Piez.: xii, 282. Type species: “*Zethus coeruleo-pennis* Fab.” [= *Vespa coeruleopennis* Fabricius, 1798].

*Zethus dolosus* Bingham, 1897

*Zethus malayanus* Gusenleitner, 2010

*Zethus nanlingensis* Nguyen & Xu, 2017

*Zethus taiwanus* Yeh & Lu, 2017

*Zehtus velamellatus* Tan, 2018

*Zethus nigerrimus* Gusenleitner, 2001
