# Corrigenda: Tan J-L, Carpenter JM, van Achterberg C (2018) An illustrated key to the genera of Eumeninae from China, with a checklist of species (Hymenoptera, Vespidae). ZooKeys 740: 109–149. https://doi.org/10.3897/zookeys.740.22654

**DOI:** 10.3897/zookeys.753.25327

**Published:** 2018-04-27

**Authors:** Jiang-Li Tan, James M. Carpenter, Cornelis van Achterberg

**Affiliations:** 1 Shaanxi Key Laboratory for Animal Conservation/Key Laboratory of Resource Biology and Biotechnology in Western China, Ministry of Education, College of Life Sciences, Northwest University, Xi’an, China; 2 Division of Invertebrate Zoology, American Museum of Natural History, New York, NY, USA

In our recently published illustrated key to the genera and checklist of the species of Chinese Eumeninae, we overlooked the paper by Kurzenko (1977). This was part of a series on the Insects of Mongolia, and contains important distributional information about Eumeninae in China. It was kindly brought to our attention by Dr A.V. Fateryga and we are glad to correct the omissions here and are grateful to use some additional comments by Dr Fateryga.

The following couplets should be changed or added to accommodate two missing genera:

**Table:** 

23	Tergum I with two transverse carinae (23a) or with one (23a’)	**24A**
–	Tergum I without transverse carinae (23aa)	**27**
24A	Propodeal dorsum without extending horizontal area (24Aa)	**24**
–	Propodeal dorsum extending horizontally, forming shelf-like area behind metanotum (24Aaa). [pretegular carina present]	**25**
24	Tegula densely punctate, sieve-like, surpassing parategula posteriorly (24a); pretegular carina absent (24b); pronotal anterior carina complete laterally, humeri angular or pointed (24c); carina of tergum I at anterior narrow part of tergum [and carina indistinct in some species] (24d)	***Jucancistrocerus* Blüthgen**
–	Tegula usually finely punctate (24aa); pretegular carina present (24bb); pronotal anterior carina partly absent laterally, humeri rounded (24cc); carina of tergum I at about middle of tergum (24dd)	***Stenancistrocerus* de Saussure**
46	Metanotum between horizontal and vertical area with hemi-circular carina (46a)	***Antodynerus* de Saussure**
–	Metanotum between areas without hemi-circular shaped carina (46aa)	46A
46A	Body mainly yellow with few black spots; pronotum anterior vertical plane usually without hyaline carina, with its anterior face densely punctate laterally (46Aa); cephalic fovea remaining far from occipital carina (46Ab)	***Chlorodynerus* Blüthgen**
–	Body mainly black with yellow patches; pronotum anterior vertical plane with hyaline carina, with its anterior face sparsely punctate laterally (46Aaa); cephalic foveae close to occipital carina (46Abb)	***Euodynerus* Dalla Torre**

**Figure 24a.**
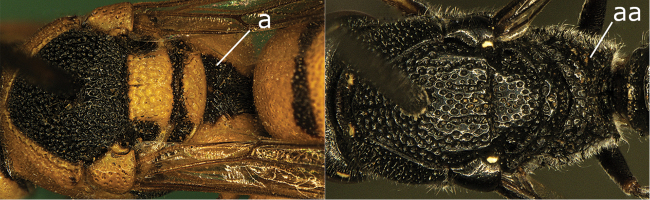
Mesosoma in dorsal view (**a, aa**). **a** Jucancistrocerus (Eremodynerus) atrofasciatus (Morawitz) **aa** *Pseudonortonia abbreviaticornis* Giordani Soika.

**Figure 24.**
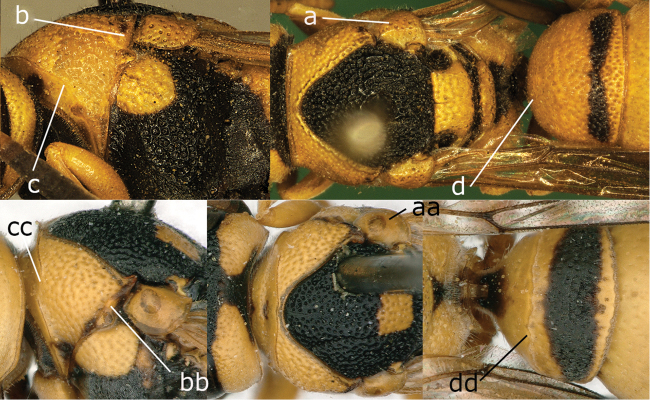
Mesosoma in lateral view (**c b, cc**, and **bb**) and in dorsal view (**a, aa**), tergum I in dorsal (**d, dd**). **a-d.** Jucancistrocerus (Eremodynerus) atrofasciatus (Morawitz); **aa-dd.** *Stenancistrocerus alluaudi* (Dusmet).

**Figure 46a.**
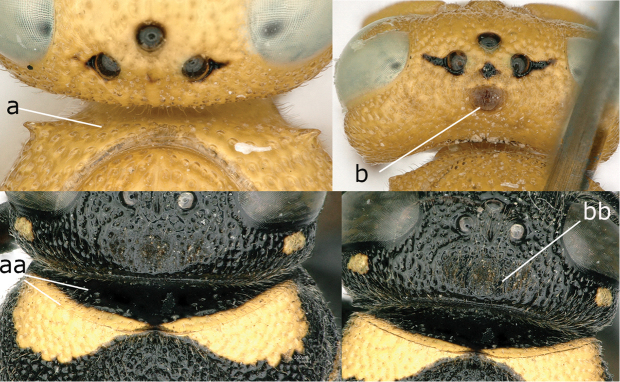
Pronotum anterior vertical plane (a, aa), head in dorsal view (b, bb). **a-b.** *Chlorodynerus diglaensis* (Blüthgen) **aa-bb.** *Euodynerus* sp.

The following species and subspecies should be added to the checklist. The omitted genera are *Cholordynerus* and *Stenancistrocerus*.

[***Ancistrocerus* Wesmael, 1836**]

*Ancistrocerus oviventris oviventrus* (Wesmael, 1836)

*Ancistrocerus raddei* (Kostylev, 1940)

*Ancistrocerus scoticus scoticus* (Curtis, 1826)

*Ancistrocerus tenellus* (Kostylev, 1935)

[***Antepipona* de Saussure, 1855**]

*Antepipona tylocifica* Kurzenko, 1977

***Chlorodynerus* Blüthgen, 1951**

*Chlorodynerus* Blüthgen, 1951, Boll. Soc. Entomol. Ital. 81: 67 (key), 75. Type species: *Odynerus chloroticus* Spinola, 1838, by original designation.

*Chlorodynerus arenicolus* (Kostylev, 1935)

[***Eumenes* Latreille, 1802**]

*Eumenes coarctatus ordubadensis* Blüthgen, 1938

*Eumenes mongolicus* Morawitz, 1889

[***Euodynerus* Dalla Torre, 1904**]

Euodynerus (Euodynerus) dantici dantici (Rossi, 1790)

Euodynerus (Euodynerus) semisaecularis macedonicus Blüthgen, 1951

[***Katamenes* Meade-Waldo, 1910**]

*Katamenes radoszkovskii* Blüthgen, 1962 (*fide* Dr A.V. Fateryga: probably a synonym of *K. dimidiatus montanus* (Nurse, 1904))

*Katamenes sichelii fulvus* (Eversmann, 1854)

[***Leptochilus* de Saussure, 1853**]

Leptochilus (Lionotulus) habyrganus Kurzenko, 1977

Leptochilus (Lionotulus) kozlovi Kurzenko, 1977

[***Onychopterocheilus* Blüthgen, 1955**]

Onychopterocheilus (Ghilarocheilus) turovi (Kostylev, 1937)

[***Pseudepipona* de Saussure, 1856**]

Pseudepipona (Deuterepipona) herzi kozlovi (Kostylev, 1937)

Pseudepipona (Pseudepipona) herrichii mongolica Giordani Soika, 1970

Pseudepipona (Pseudepipona) kozhevnikovi (Kostylev, 1927)

Pseudepipona (Pseudepipona) tricarinata (Kokujev, 1912)

**Note.** The species listed in the checklist belong all to the subgenus Pseudepipona de Saussure.

[***Pterocheilus* Klug, 1805**]

Pterocheilus (Pterocheilus) heptneri Kostylev, 1940

Pterocheilus (Pterocheilus) mandibularis Morawitz, 1889

Pterocheilus (Pterocheilus) quaesitus (Morawitz, 1895)

Pterocheilus (Pterocheilus) sibiricus sibiricus (Morawitz, 1867)

***Stenancistrocerus* de Saussure, 1863**

*Stenancistrocerus* de Saussure, 1863, Mém. Soc. Phys. Hist. Nat. Genève 17 (1): 216. Type species: *Odynerus atropos* Lepeletier, 1841.

Stenancistrocerus (Paratropancistrocerus) transcaspicus (Kostylev, 1935)

[***Symmorphus* Wesmael, 1836**]

*Symmorphus allobrogus* (de Saussure, 1855) (Dr A.V. Fateryga *in litt*.: reported as *S. bifasciatus* in Kurzenko (1977); true *S. bifasciatus* was reported as *S. mutinensis*)

*Symmorphus crassicornis* (Panzer, 1798)

**Correction** (by Dr A.V. Fateryga):

*Brachyodynerus perrarus* (not “*perarrus*”) Kurzenko, 1977

## References

[B1] Kurzenko NV (1977) Eumenid wasps (Hymenoptera, Eumenidae) of the Mogolian People’s Republic and adjacent regions of China and Southern Siberia. Nasekomye Mongolii [Insects of Mongolia] 5: 537–582. [In Russian]

